# Measurements of the $$\mathrm{Z}$$$$\mathrm{Z}$$ production cross sections in the $$2\mathrm{l} 2\nu $$ channel in proton–proton collisions at $$\sqrt{s} = 7$$ and $$8~\mathrm{TeV} $$ and combined constraints on triple gauge couplings

**DOI:** 10.1140/epjc/s10052-015-3706-0

**Published:** 2015-10-29

**Authors:** V. Khachatryan, A. M. Sirunyan, A. Tumasyan, W. Adam, T. Bergauer, M. Dragicevic, J. Erö, M. Friedl, R. Frühwirth, V. M. Ghete, C. Hartl, N. Hörmann, J. Hrubec, M. Jeitler, W. Kiesenhofer, V. Knünz, M. Krammer, I. Krätschmer, D. Liko, I. Mikulec, D. Rabady, B. Rahbaran, H. Rohringer, R. Schöfbeck, J. Strauss, W. Treberer-Treberspurg, W. Waltenberger, C.-E. Wulz, V. Mossolov, N. Shumeiko, J. Suarez Gonzalez, S. Alderweireldt, S. Bansal, T. Cornelis, E. A. De Wolf, X. Janssen, A. Knutsson, J. Lauwers, S. Luyckx, S. Ochesanu, R. Rougny, M. Van De Klundert, H. Van Haevermaet, P. Van Mechelen, N. Van Remortel, A. Van Spilbeeck, F. Blekman, S. Blyweert, J. D’Hondt, N. Daci, N. Heracleous, J. Keaveney, S. Lowette, M. Maes, A. Olbrechts, Q. Python, D. Strom, S. Tavernier, W. Van Doninck, P. Van Mulders, G. P. Van Onsem, I. Villella, C. Caillol, B. Clerbaux, G. De Lentdecker, D. Dobur, L. Favart, A. P. R. Gay, A. Grebenyuk, A. Léonard, A. Mohammadi, L. Perniè, A. Randle-conde, T. Reis, T. Seva, L. Thomas, C. Vander Velde, P. Vanlaer, J. Wang, F. Zenoni, V. Adler, K. Beernaert, L. Benucci, A. Cimmino, S. Costantini, S. Crucy, S. Dildick, A. Fagot, G. Garcia, J. Mccartin, A. A. Ocampo Rios, D. Ryckbosch, S. Salva Diblen, M. Sigamani, N. Strobbe, F. Thyssen, M. Tytgat, E. Yazgan, N. Zaganidis, S. Basegmez, C. Beluffi, G. Bruno, R. Castello, A. Caudron, L. Ceard, G. G. Da Silveira, C. Delaere, T. du Pree, D. Favart, L. Forthomme, A. Giammanco, J. Hollar, A. Jafari, P. Jez, M. Komm, V. Lemaitre, C. Nuttens, D. Pagano, L. Perrini, A. Pin, K. Piotrzkowski, A. Popov, L. Quertenmont, M. Selvaggi, M. Vidal Marono, J. M. Vizan Garcia, N. Beliy, T. Caebergs, E. Daubie, G. H. Hammad, W. L. Aldá Júnior, G. A. Alves, L. Brito, M. Correa Martins Junior, T. Dos Reis Martins, C. Mora Herrera, M. E. Pol, P. Rebello Teles, W. Carvalho, J. Chinellato, A. Custódio, E. M. Da Costa, D. De Jesus Damiao, C. De Oliveira Martins, S. Fonseca De Souza, H. Malbouisson, D. Matos Figueiredo, L. Mundim, H. Nogima, W. L. Prado Da Silva, J. Santaolalla, A. Santoro, A. Sznajder, E. J. Tonelli Manganote, A. Vilela Pereira, C. A. Bernardes, S. Dogra, T. R. Fernandez Perez Tomei, E. M. Gregores, P. G. Mercadante, S. F. Novaes, Sandra S. Padula, A. Aleksandrov, V. Genchev, R. Hadjiiska, P. Iaydjiev, A. Marinov, S. Piperov, M. Rodozov, G. Sultanov, M. Vutova, A. Dimitrov, I. Glushkov, L. Litov, B. Pavlov, P. Petkov, J. G. Bian, G. M. Chen, H. S. Chen, M. Chen, T. Cheng, R. Du, C. H. Jiang, R. Plestina, F. Romeo, J. Tao, Z. Wang, C. Asawatangtrakuldee, Y. Ban, Q. Li, S. Liu, Y. Mao, S. J. Qian, D. Wang, Z. Xu, W. Zou, C. Avila, A. Cabrera, L. F. Chaparro Sierra, C. Florez, J. P. Gomez, B. Gomez Moreno, J. C. Sanabria, N. Godinovic, D. Lelas, D. Polic, I. Puljak, Z. Antunovic, M. Kovac, V. Brigljevic, K. Kadija, J. Luetic, D. Mekterovic, L. Sudic, A. Attikis, G. Mavromanolakis, J. Mousa, C. Nicolaou, F. Ptochos, P. A. Razis, M. Bodlak, M. Finger, M. Finger, Y. Assran, A. Ellithi Kamel, M. A. Mahmoud, A. Radi, M. Kadastik, M. Murumaa, M. Raidal, A. Tiko, P. Eerola, G. Fedi, M. Voutilainen, J. Härkönen, V. Karimäki, R. Kinnunen, M. J. Kortelainen, T. Lampén, K. Lassila-Perini, S. Lehti, T. Lindén, P. Luukka, T. Mäenpää, T. Peltola, E. Tuominen, J. Tuominiemi, E. Tuovinen, L. Wendland, J. Talvitie, T. Tuuva, M. Besancon, F. Couderc, M. Dejardin, D. Denegri, B. Fabbro, J. L. Faure, C. Favaro, F. Ferri, S. Ganjour, A. Givernaud, P. Gras, G. Hamel de Monchenault, P. Jarry, E. Locci, J. Malcles, J. Rander, A. Rosowsky, M. Titov, S. Baffioni, F. Beaudette, P. Busson, C. Charlot, T. Dahms, M. Dalchenko, L. Dobrzynski, N. Filipovic, A. Florent, R. Granier de Cassagnac, L. Mastrolorenzo, P. Miné, C. Mironov, I. N. Naranjo, M. Nguyen, C. Ochando, G. Ortona, P. Paganini, S. Regnard, R. Salerno, J. B. Sauvan, Y. Sirois, C. Veelken, Y. Yilmaz, A. Zabi, J.-L. Agram, J. Andrea, A. Aubin, D. Bloch, J.-M. Brom, E. C. Chabert, C. Collard, E. Conte, J.-C. Fontaine, D. Gelé, U. Goerlach, C. Goetzmann, A.-C. Le Bihan, K. Skovpen, P. Van Hove, S. Gadrat, S. Beauceron, N. Beaupere, C. Bernet, G. Boudoul, E. Bouvier, S. Brochet, C. A. Carrillo Montoya, J. Chasserat, R. Chierici, D. Contardo, P. Depasse, H. El Mamouni, J. Fan, J. Fay, S. Gascon, M. Gouzevitch, B. Ille, T. Kurca, M. Lethuillier, L. Mirabito, S. Perries, J. D. Ruiz Alvarez, D. Sabes, L. Sgandurra, V. Sordini, M. Vander Donckt, P. Verdier, S. Viret, H. Xiao, Z. Tsamalaidze, C. Autermann, S. Beranek, M. Bontenackels, M. Edelhoff, L. Feld, A. Heister, O. Hindrichs, K. Klein, A. Ostapchuk, M. Preuten, F. Raupach, J. Sammet, S. Schael, J. F. Schulte, H. Weber, B. Wittmer, V. Zhukov, M. Ata, M. Brodski, E. Dietz-Laursonn, D. Duchardt, M. Erdmann, R. Fischer, A. Güth, T. Hebbeker, C. Heidemann, K. Hoepfner, D. Klingebiel, S. Knutzen, P. Kreuzer, M. Merschmeyer, A. Meyer, P. Millet, M. Olschewski, K. Padeken, P. Papacz, H. Reithler, S. A. Schmitz, L. Sonnenschein, D. Teyssier, S. Thüer, M. Weber, V. Cherepanov, Y. Erdogan, G. Flügge, H. Geenen, M. Geisler, W. Haj Ahmad, F. Hoehle, B. Kargoll, T. Kress, Y. Kuessel, A. Künsken, J. Lingemann, A. Nowack, I. M. Nugent, O. Pooth, A. Stahl, M. Aldaya Martin, I. Asin, N. Bartosik, J. Behr, U. Behrens, A. J. Bell, A. Bethani, K. Borras, A. Burgmeier, A. Cakir, L. Calligaris, A. Campbell, S. Choudhury, F. Costanza, C. Diez Pardos, G. Dolinska, S. Dooling, T. Dorland, G. Eckerlin, D. Eckstein, T. Eichhorn, G. Flucke, J. Garay Garcia, A. Geiser, P. Gunnellini, J. Hauk, M. Hempel, H. Jung, A. Kalogeropoulos, M. Kasemann, P. Katsas, J. Kieseler, C. Kleinwort, l. Korol, D. Krücker, W. Lange, J. Leonard, K. Lipka, A. Lobanov, W. Lohmann, B. Lutz, R. Mankel, I. Marfin, I.-A. Melzer-Pellmann, A. B. Meyer, G. Mittag, J. Mnich, A. Mussgiller, S. Naumann-Emme, A. Nayak, E. Ntomari, H. Perrey, D. Pitzl, R. Placakyte, A. Raspereza, P. M. Ribeiro Cipriano, B. Roland, E. Ron, M. Ö. Sahin, J. Salfeld-Nebgen, P. Saxena, T. Schoerner-Sadenius, M. Schröder, C. Seitz, S. Spannagel, A. D. R. Vargas Trevino, R. Walsh, C. Wissing, V. Blobel, M. Centis Vignali, A. R. Draeger, J. Erfle, E. Garutti, K. Goebel, M. Görner, J. Haller, M. Hoffmann, R. S. Höing, A. Junkes, H. Kirschenmann, R. Klanner, R. Kogler, J. Lange, T. Lapsien, T. Lenz, I. Marchesini, J. Ott, T. Peiffer, A. Perieanu, N. Pietsch, J. Poehlsen, T. Poehlsen, D. Rathjens, C. Sander, H. Schettler, P. Schleper, E. Schlieckau, A. Schmidt, M. Seidel, V. Sola, H. Stadie, G. Steinbrück, D. Troendle, E. Usai, L. Vanelderen, A. Vanhoefer, C. Barth, C. Baus, J. Berger, C. Böser, E. Butz, T. Chwalek, W. De Boer, A. Descroix, A. Dierlamm, M. Feindt, F. Frensch, M. Giffels, A. Gilbert, F. Hartmann, T. Hauth, U. Husemann, I. Katkov, A. Kornmayer, E. Kuznetsova, P. Lobelle Pardo, M. U. Mozer, T. Müller, Th. Müller, A. Nürnberg, G. Quast, K. Rabbertz, S. Röcker, H. J. Simonis, F. M. Stober, R. Ulrich, J. Wagner-Kuhr, S. Wayand, T. Weiler, R. Wolf, G. Anagnostou, G. Daskalakis, T. Geralis, V. A. Giakoumopoulou, A. Kyriakis, D. Loukas, A. Markou, C. Markou, A. Psallidas, I. Topsis-Giotis, A. Agapitos, S. Kesisoglou, A. Panagiotou, N. Saoulidou, E. Stiliaris, X. Aslanoglou, I. Evangelou, G. Flouris, C. Foudas, P. Kokkas, N. Manthos, I. Papadopoulos, E. Paradas, J. Strologas, G. Bencze, C. Hajdu, P. Hidas, D. Horvath, F. Sikler, V. Veszpremi, G. Vesztergombi, A. J. Zsigmond, N. Beni, S. Czellar, J. Karancsi, J. Molnar, J. Palinkas, Z. Szillasi, A. Makovec, P. Raics, Z. L. Trocsanyi, B. Ujvari, S. K. Swain, S. B. Beri, V. Bhatnagar, R. Gupta, U. Bhawandeep, A. K. Kalsi, M. Kaur, R. Kumar, M. Mittal, N. Nishu, J. B. Singh, Ashok Kumar, Arun Kumar, S. Ahuja, A. Bhardwaj, B. C. Choudhary, A. Kumar, S. Malhotra, M. Naimuddin, K. Ranjan, V. Sharma, S. Banerjee, S. Bhattacharya, K. Chatterjee, S. Dutta, B. Gomber, Sa. Jain, Sh. Jain, R. Khurana, A. Modak, S. Mukherjee, D. Roy, S. Sarkar, M. Sharan, A. Abdulsalam, D. Dutta, V. Kumar, A. K. Mohanty, L. M. Pant, P. Shukla, A. Topkar, T. Aziz, S. Banerjee, S. Bhowmik, R. M. Chatterjee, R. K. Dewanjee, S. Dugad, S. Ganguly, S. Ghosh, M. Guchait, A. Gurtu, G. Kole, S. Kumar, M. Maity, G. Majumder, K. Mazumdar, G. B. Mohanty, B. Parida, K. Sudhakar, N. Wickramage, H. Bakhshiansohi, H. Behnamian, S. M. Etesami, A. Fahim, R. Goldouzian, M. Khakzad, M. Mohammadi Najafabadi, M. Naseri, S. Paktinat Mehdiabadi, F. Rezaei Hosseinabadi, B. Safarzadeh, M. Zeinali, M. Felcini, M. Grunewald, M. Abbrescia, C. Calabria, S. S. Chhibra, A. Colaleo, D. Creanza, N. De Filippis, M. De Palma, L. Fiore, G. Iaselli, G. Maggi, M. Maggi, S. My, S. Nuzzo, A. Pompili, G. Pugliese, R. Radogna, G. Selvaggi, A. Sharma, L. Silvestris, R. Venditti, P. Verwilligen, G. Abbiendi, A. C. Benvenuti, D. Bonacorsi, S. Braibant-Giacomelli, L. Brigliadori, R. Campanini, P. Capiluppi, A. Castro, F. R. Cavallo, G. Codispoti, M. Cuffiani, G. M. Dallavalle, F. Fabbri, A. Fanfani, D. Fasanella, P. Giacomelli, C. Grandi, L. Guiducci, S. Marcellini, G. Masetti, A. Montanari, F. L. Navarria, A. Perrotta, F. Primavera, A. M. Rossi, F. Primavera, T. Rovelli, G. P. Siroli, N. Tosi, R. Travaglini, S. Albergo, G. Cappello, M. Chiorboli, S. Costa, F. Giordano, R. Potenza, A. Tricomi, C. Tuve, G. Barbagli, V. Ciulli, C. Civinini, R. D’Alessandro, E. Focardi, E. Gallo, S. Gonzi, V. Gori, P. Lenzi, M. Meschini, S. Paoletti, G. Sguazzoni, A. Tropiano, L. Benussi, S. Bianco, F. Fabbri, D. Piccolo, R. Ferretti, F. Ferro, M. Lo Vetere, E. Robutti, S. Tosi, M. E. Dinardo, S. Fiorendi, S. Gennai, R. Gerosa, A. Ghezzi, P. Govoni, M. T. Lucchini, S. Malvezzi, R. A. Manzoni, A. Martelli, B. Marzocchi, D. Menasce, L. Moroni, M. Paganoni, D. Pedrini, S. Ragazzi, N. Redaelli, T. Tabarelli de Fatis, S. Buontempo, N. Cavallo, S. Di Guida, F. Fabozzi, A. O. M. Iorio, L. Lista, S. Meola, M. Merola, P. Paolucci, P. Azzi, N. Bacchetta, D. Bisello, R. Carlin, P. Checchia, M. Dall’Osso, T. Dorigo, U. Dosselli, F. Gasparini, U. Gasparini, A. Gozzelino, K. Kanishchev, S. Lacaprara, M. Margoni, A. T. Meneguzzo, M. Passaseo, J. Pazzini, N. Pozzobon, P. Ronchese, F. Simonetto, E. Torassa, M. Tosi, P. Zotto, A. Zucchetta, G. Zumerle, M. Gabusi, S. P. Ratti, V. Re, C. Riccardi, P. Salvini, P. Vitulo, M. Biasini, G. M. Bilei, D. Ciangottini, L. Fanò, P. Lariccia, G. Mantovani, M. Menichelli, A. Saha, A. Santocchia, A. Spiezia, K. Androsov, P. Azzurri, G. Bagliesi, J. Bernardini, T. Boccali, G. Broccolo, R. Castaldi, M. A. Ciocci, R. Dell’Orso, S. Donato, G. Fedi, F. Fiori, L. Foà, A. Giassi, M. T. Grippo, F. Ligabue, T. Lomtadze, L. Martini, A. Messineo, C. S. Moon, F. Palla, A. Rizzi, A. Savoy-Navarro, A. T. Serban, P. Spagnolo, P. Squillacioti, R. Tenchini, G. Tonelli, A. Venturi, P. G. Verdini, C. Vernieri, L. Barone, F. Cavallari, G. D’imperio, D. Del Re, M. Diemoz, C. Jorda, E. Longo, F. Margaroli, P. Meridiani, F. Micheli, G. Organtini, R. Paramatti, S. Rahatlou, C. Rovelli, F. Santanastasio, L. Soffi, P. Traczyk, N. Amapane, R. Arcidiacono, S. Argiro, M. Arneodo, R. Bellan, C. Biino, N. Cartiglia, S. Casasso, M. Costa, R. Covarelli, A. Degano, N. Demaria, L. Finco, C. Mariotti, S. Maselli, E. Migliore, V. Monaco, M. Musich, M. M. Obertino, L. Pacher, N. Pastrone, M. Pelliccioni, G. L. Pinna Angioni, A. Potenza, A. Romero, M. Ruspa, R. Sacchi, A. Solano, A. Staiano, U. Tamponi, S. Belforte, V. Candelise, M. Casarsa, F. Cossutti, G. Della Ricca, B. Gobbo, C. La Licata, M. Marone, A. Schizzi, T. Umer, A. Zanetti, S. Chang, T. A. Kropivnitskaya, S. K. Nam, D. H. Kim, G. N. Kim, M. S. Kim, M. S. Kim, D. J. Kong, S. Lee, Y. D. Oh, H. Park, A. Sakharov, D. C. Son, T. J. Kim, M. S. Ryn, J. Y. Kim, D. H. Moon, S. Song, S. Choi, D. Gyun, B. Hong, M. Jo, H. Kim, Y. Kim, B. Lee, K. S. Lee, S. K. Park, Y. Roh, H. D. Yoo, M. Choi, J. H. Kim, I. C. Park, G. Ryu, Y. Choi, Y. K. Choi, J. Goh, D. Kim, E. Kwon, J. Lee, I. Yu, A. Juodagalvis, J. R. Komaragiri, M. A. B. Md Ali, W. A. T. Wan Abdullah, E. Casimiro Linares, H. Castilla-Valdez, E. De La Cruz-Burelo, I. Heredia-de La Cruz, A. Hernandez-Almada, R. Lopez-Fernandez, A. Sanchez-Hernandez, S. Carrillo Moreno, F. Vazquez Valencia, I. Pedraza, H. A. Salazar Ibarguen, A. Morelos Pineda, D. Krofcheck, P. H. Butler, S. Reucroft, A. Ahmad, M. Ahmad, Q. Hassan, H. R. Hoorani, W. A. Khan, T. Khurshid, M. Shoaib, H. Bialkowska, M. Bluj, B. Boimska, T. Frueboes, M. Górski, M. Kazana, K. Nawrocki, K. Romanowska-Rybinska, M. Szleper, P. Zalewski, G. Brona, K. Bunkowski, M. Cwiok, W. Dominik, K. Doroba, A. Kalinowski, M. Konecki, J. Krolikowski, M. Misiura, M. Olszewski, P. Bargassa, C. Beir ao Da Cruz E Silva, P. Faccioli, P. G. Ferreira Parracho, M. Gallinaro, L. Lloret Iglesias, F. Nguyen, J. Rodrigues Antunes, J. Seixas, J. Varela, P. Vischia, S. Afanasiev, P. Bunin, I. Golutvin, A. Kamenev, V. Karjavin, V. Konoplyanikov, G. Kozlov, A. Lanev, A. Malakhov, V. Matveev, P. Moisenz, V. Palichik, V. Perelygin, M. Savina, S. Shmatov, S. Shulha, V. Smirnov, A. Zarubin, V. Golovtsov, Y. Ivanov, V. Kim, E. Kuznetsova, P. Levchenko, V. Murzin, V. Oreshkin, I. Smirnov, V. Sulimov, L. Uvarov, S. Vavilov, A. Vorobyev, An. Vorobyev, Yu. Andreev, A. Dermenev, S. Gninenko, N. Golubev, M. Kirsanov, N. Krasnikov, A. Pashenkov, D. Tlisov, A. Toropin, V. Epshteyn, V. Gavrilov, N. Lychkovskaya, V. Popov, l. Pozdnyakov, G. Safronov, S. Semenov, A. Spiridonov, V. Stolin, E. Vlasov, A. Zhokin, V. Andreev, M. Azarkin, I. Dremin, M. Kirakosyan, A. Leonidov, G. Mesyats, S. V. Rusakov, A. Vinogradov, A. Belyaev, E. Boos, M. Dubinin, L. Dudko, A. Ershov, A. Gribushin, V. Klyukhin, O. Kodolova, I. Lokhtin, S. Obraztsov, S. Petrushanko, V. Savrin, A. Snigirev, I. Azhgirey, I. Bayshev, S. Bitioukov, V. Kachanov, A. Kalinin, D. Konstantinov, V. Krychkine, V. Petrov, R. Ryutin, A. Sobol, L. Tourtchanovitch, S. Troshin, N. Tyurin, A. Uzunian, A. Volkov, P. Adzic, M. Ekmedzic, J. Milosevic, V. Rekovic, J. Alcaraz Maestre, C. Battilana, E. Calvo, M. Cerrada, M. Chamizo Llatas, N. Colino, B. De La Cruz, A. Delgado Peris, D. Domínguez Vázquez, A. Escalante Del Valle, C. Fernandez Bedoya, J. P. Fernández Ramos, J. Flix, M. C. Fouz, P. Garcia-Abia, O. Gonzalez Lopez, S. Goy Lopez, J. M. Hernandez, M. I. Josa, E. Navarro De Martino, A. Pérez-Calero Yzquierdo, J. Puerta Pelayo, A. Quintario Olmeda, I. Redondo, L. Romero, M. S. Soares, C. Albajar, J. F. de Trocóniz, M. Missiroli, D. Moran, H. Brun, J. Cuevas, J. Fernandez Menendez, S. Folgueras, I. Gonzalez Caballero, J. A. Brochero Cifuentes, I. J. Cabrillo, A. Calderon, J. Duarte Campderros, M. Fernandez, G. Gomez, A. Graziano, A. Lopez Virto, J. Marco, R. Marco, C. Martinez Rivero, F. Matorras, F. J. Munoz Sanchez, J. Piedra Gomez, T. Rodrigo, A. Y. Rodríguez-Marrero, A. Ruiz-Jimeno, L. Scodellaro, I. Vila, R. Vilar Cortabitarte, D. Abbaneo, E. Auffray, G. Auzinger, M. Bachtis, P. Baillon, A. H. Ball, D. Barney, A. Benaglia, J. Bendavid, L. Benhabib, J. F. Benitez, P. Bloch, A. Bocci, A. Bonato, O. Bondu, C. Botta, H. Breuker, T. Camporesi, G. Cerminara, S. Colafranceschi, M. D’Alfonso, D. d’Enterria, A. Dabrowski, A. David, F. De Guio, A. De Roeck, S. De Visscher, E. Di Marco, M. Dobson, M. Dordevic, B. Dorney, N. Dupont-Sagorin, A. Elliott-Peisert, G. Franzoni, W. Funk, D. Gigi, K. Gill, D. Giordano, M. Girone, F. Glege, R. Guida, S. Gundacker, M. Guthoff, J. Hammer, M. Hansen, P. Harris, J. Hegeman, V. Innocente, P. Janot, K. Kousouris, K. Krajczar, P. Lecoq, C. Lourenço, N. Magini, L. Malgeri, M. Mannelli, J. Marrouche, L. Masetti, F. Meijers, S. Mersi, E. Meschi, F. Moortgat, S. Morovic, M. Mulders, L. Orsini, L. Pape, E. Perez, A. Petrilli, G. Petrucciani, A. Pfeiffer, M. Pimiä, D. Piparo, M. Plagge, A. Racz, G. Rolandi, M. Rovere, H. Sakulin, C. Schäfer, C. Schwick, A. Sharma, P. Siegrist, P. Silva, M. Simon, P. Sphicas, D. Spiga, J. Steggemann, B. Stieger, M. Stoye, Y. Takahashi, D. Treille, A. Tsirou, G. I. Veres, N. Wardle, H. K. Wöhri, H. Wollny, W. D. Zeuner, W. Bertl, K. Deiters, W. Erdmann, R. Horisberger, Q. Ingram, H. C. Kaestli, D. Kotlinski, U. Langenegger, D. Renker, T. Rohe, F. Bachmair, L. Bäni, L. Bianchini, M. A. Buchmann, B. Casal, N. Chanon, G. Dissertori, M. Dittmar, M. Donegà, M. Dünser, P. Eller, C. Grab, D. Hits, J. Hoss, W. Lustermann, B. Mangano, A. C. Marini, M. Marionneau, P. Martinez Ruiz del Arbol, M. Masciovecchio, D. Meister, N. Mohr, P. Musella, C. Nägeli, F. Nessi-Tedaldi, F. Pandolfi, F. Pauss, L. Perrozzi, M. Peruzzi, M. Quittnat, L. Rebane, M. Rossini, A. Starodumov, M. Takahashi, K. Theofilatos, R. Wallny, H. A. Weber, C. Amsler, M. F. Canelli, V. Chiochia, A. De Cosa, A. Hinzmann, T. Hreus, B. Kilminster, C. Lange, J. Ngadiuba, D. Pinna, P. Robmann, F. J. Ronga, S. Taroni, Y. Yang, M. Cardaci, K. H. Chen, C. Ferro, C. M. Kuo, W. Lin, Y. J. Lu, R. Volpe, S. S. Yu, P. Chang, Y. H. Chang, Y. Chao, K. F. Chen, P. H. Chen, C. Dietz, U. Grundler, W.-S. Hou, Y. F. Liu, R.-S. Lu, M. Miñano Moya, E. Petrakou, Y. M. Tzeng, R. Wilken, B. Asavapibhop, G. Singh, N. Srimanobhas, N. Suwonjandee, A. Adiguzel, M. N. Bakirci, S. Cerci, C. Dozen, I. Dumanoglu, E. Eskut, S. Girgis, G. Gokbulut, Y. Guler, E. Gurpinar, I. Hos, E. E. Kangal, A. Kayis Topaksu, G. Onengut, K. Ozdemir, S. Ozturk, A. Polatoz, D. Sunar Cerci, B. Tali, H. Topakli, M. Vergili, C. Zorbilmez, I. V. Akin, B. Bilin, S. Bilmis, H. Gamsizkan, B. Isildak, G. Karapinar, K. Ocalan, S. Sekmen, U. E. Surat, M. Yalvac, M. Zeyrek, A. Albayrak, E. Gülmez, M. Kaya, O. Kaya, T. Yetkin, K. Cankocak, F. I. Vardarlı, L. Levchuk, P. Sorokin, J. J. Brooke, E. Clement, D. Cussans, H. Flacher, J. Goldstein, M. Grimes, G. P. Heath, H. F. Heath, J. Jacob, L. Kreczko, C. Lucas, Z. Meng, D. M. Newbold, S. Paramesvaran, A. Poll, T. Sakuma, S. Seif El Nasr-storey, S. Senkin, V. J. Smith, K. W. Bell, A. Belyaev, C. Brew, R. M. Brown, D. J. A. Cockerill, J. A. Coughlan, K. Harder, S. Harper, E. Olaiya, D. Petyt, C. H. Shepherd-Themistocleous, A. Thea, I. R. Tomalin, T. Williams, W. J. Womersley, S. D. Worm, M. Baber, R. Bainbridge, O. Buchmuller, D. Burton, D. Colling, N. Cripps, P. Dauncey, G. Davies, M. Della Negra, P. Dunne, A. Elwood, W. Ferguson, J. Fulcher, D. Futyan, G. Hall, G. Iles, M. Jarvis, G. Karapostoli, M. Kenzie, R. Lane, R. Lucas, L. Lyons, A.-M. Magnan, S. Malik, B. Mathias, J. Nash, A. Nikitenko, J. Pela, M. Pesaresi, K. Petridis, D. M. Raymond, S. Rogerson, A. Rose, C. Seez, P. Sharp, A. Tapper, M. Vazquez Acosta, T. Virdee, S. C. Zenz, J. E. Cole, P. R. Hobson, A. Khan, P. Kyberd, D. Leggat, D. Leslie, I. D. Reid, P. Symonds, L. Teodorescu, M. Turner, J. Dittmann, K. Hatakeyama, A. Kasmi, H. Liu, N. Pastika, T. Scarborough, Z. Wu, O. Charaf, S. I. Cooper, C. Henderson, P. Rumerio, A. Avetisyan, T. Bose, C. Fantasia, P. Lawson, C. Richardson, J. Rohlf, J. St. John, L. Sulak, J. Alimena, E. Berry, S. Bhattacharya, G. Christopher, D. Cutts, Z. Demiragli, N. Dhingra, A. Ferapontov, A. Garabedian, U. Heintz, E. Laird, G. Landsberg, M. Narain, S. Sagir, T. Sinthuprasith, T. Speer, J. Swanson, R. Breedon, G. Breto, M. Calderon De La Barca Sanchez, S. Chauhan, M. Chertok, J. Conway, R. Conway, P. T. Cox, R. Erbacher, M. Gardner, W. Ko, R. Lander, M. Mulhearn, D. Pellett, J. Pilot, F. Ricci-Tam, S. Shalhout, J. Smith, M. Squires, D. Stolp, M. Tripathi, S. Wilbur, R. Yohay, R. Cousins, P. Everaerts, C. Farrell, J. Hauser, M. Ignatenko, G. Rakness, E. Takasugi, V. Valuev, M. Weber, K. Burt, R. Clare, J. Ellison, J. W. Gary, G. Hanson, J. Heilman, M. Ivova Rikova, P. Jandir, E. Kennedy, F. Lacroix, O. R. Long, A. Luthra, M. Malberti, M. Olmedo Negrete, A. Shrinivas, S. Sumowidagdo, S. Wimpenny, J. G. Branson, G. B. Cerati, S. Cittolin, R. T. D’Agnolo, A. Holzner, R. Kelley, D. Klein, J. Letts, I. Macneill, D. Olivito, S. Padhi, C. Palmer, M. Pieri, M. Sani, V. Sharma, S. Simon, M. Tadel, Y. Tu, A. Vartak, C. Welke, F. Würthwein, A. Yagil, G. Zevi Della Porta, D. Barge, J. Bradmiller-Feld, C. Campagnari, T. Danielson, A. Dishaw, V. Dutta, K. Flowers, M. Franco Sevilla, P. Geffert, C. George, F. Golf, L. Gouskos, J. Incandela, C. Justus, N. Mccoll, S. D. Mullin, J. Richman, D. Stuart, W. To, C. West, J. Yoo, A. Apresyan, A. Bornheim, J. Bunn, Y. Chen, J. Duarte, A. Mott, H. B. Newman, C. Pena, M. Pierini, M. Spiropulu, J. R. Vlimant, R. Wilkinson, S. Xie, R. Y. Zhu, V. Azzolini, A. Calamba, B. Carlson, T. Ferguson, Y. Iiyama, M. Paulini, J. Russ, H. Vogel, I. Vorobiev, J. P. Cumalat, W. T. Ford, A. Gaz, M. Krohn, E. Luiggi Lopez, U. Nauenberg, J. G. Smith, K. Stenson, S. R. Wagner, J. Alexander, A. Chatterjee, J. Chaves, J. Chu, S. Dittmer, N. Eggert, N. Mirman, G. Nicolas Kaufman, J. R. Patterson, A. Ryd, E. Salvati, L. Skinnari, W. Sun, W. D. Teo, J. Thom, J. Thompson, J. Tucker, Y. Weng, L. Winstrom, P. Wittich, D. Winn, S. Abdullin, M. Albrow, J. Anderson, G. Apollinari, L. A. T. Bauerdick, A. Beretvas, J. Berryhill, P. C. Bhat, G. Bolla, K. Burkett, J. N. Butler, H. W. K. Cheung, F. Chlebana, S. Cihangir, V. D. Elvira, I. Fisk, J. Freeman, E. Gottschalk, L. Gray, D. Green, S. Grünendahl, O. Gutsche, J. Hanlon, D. Hare, R. M. Harris, J. Hirschauer, B. Hooberman, S. Jindariani, M. Johnson, U. Joshi, B. Klima, B. Kreis, S. Kwan, J. Linacre, D. Lincoln, R. Lipton, T. Liu, J. Lykken, K. Maeshima, J. M. Marraffino, V. I. Martinez Outschoorn, S. Maruyama, D. Mason, P. McBride, P. Merkel, K. Mishra, S. Mrenna, S. Nahn, C. Newman-Holmes, V. O’Dell, O. Prokofyev, E. Sexton-Kennedy, A. Soha, W. J. Spalding, L. Spiegel, L. Taylor, S. Tkaczyk, N. V. Tran, L. Uplegger, E. W. Vaandering, R. Vidal, A. Whitbeck, J. Whitmore, F. Yang, D. Acosta, P. Avery, P. Bortignon, D. Bourilkov, M. Carver, D. Curry, S. Das, M. De Gruttola, G. P. Di Giovanni, R. D. Field, M. Fisher, I. K. Furic, J. Hugon, J. Konigsberg, A. Korytov, T. Kypreos, J. F. Low, K. Matchev, H. Mei, P. Milenovic, G. Mitselmakher, L. Muniz, A. Rinkevicius, L. Shchutska, M. Snowball, D. Sperka, J. Yelton, M. Zakaria, S. Hewamanage, S. Linn, P. Markowitz, G. Martinez, J. L. Rodriguez, J. R. Adams, T. Adams, A. Askew, J. Bochenek, B. Diamond, J. Haas, S. Hagopian, V. Hagopian, K. F. Johnson, H. Prosper, V. Veeraraghavan, M. Weinberg, M. M. Baarmand, M. Hohlmann, H. Kalakhety, F. Yumiceva, M. R. Adams, L. Apanasevich, D. Berry, R. R. Betts, I. Bucinskaite, R. Cavanaugh, O. Evdokimov, L. Gauthier, C. E. Gerber, D. J. Hofman, P. Kurt, C. O’Brien, l. D. Sandoval Gonzalez, C. Silkworth, P. Turner, N. Varelas, B. Bilki, W. Clarida, K. Dilsiz, M. Haytmyradov, J.-P. Merlo, H. Mermerkaya, A. Mestvirishvili, A. Moeller, J. Nachtman, H. Ogul, Y. Onel, F. Ozok, A. Penzo, R. Rahmat, S. Sen, P. Tan, E. Tiras, J. Wetzel, K. Yi, I. Anderson, B. A. Barnett, B. Blumenfeld, S. Bolognesi, D. Fehling, A. V. Gritsan, P. Maksimovic, C. Martin, M. Swartz, M. Xiao, P. Baringer, A. Bean, G. Benelli, C. Bruner, J. Gray, R. P. Kenny, D. Majumder, M. Malek, M. Murray, D. Noonan, S. Sanders, J. Sekaric, R. Stringer, Q. Wang, J. S. Wood, I. Chakaberia, A. Ivanov, K. Kaadze, S. Khalil, M. Makouski, Y. Maravin, L. K. Saini, N. Skhirtladze, I. Svintradze, J. Gronberg, D. Lange, F. Rebassoo, D. Wright, A. Baden, A. Belloni, B. Calvert, S. C. Eno, J. A. Gomez, N. J. Hadley, S. Jabeen, R. G. Kellogg, T. Kolberg, Y. Lu, A. C. Mignerey, K. Pedro, A. Skuja, M. B. Tonjes, S. C. Tonwar, A. Apyan, R. Barbieri, K. Bierwagen, W. Busza, I. A. Cali, L. Di Matteo, G. Gomez Ceballos, M. Goncharov, D. Gulhan, M. Klute, Y. S. Lai, Y.-J. Lee, A. Levin, P. D. Luckey, C. Paus, D. Ralph, C. Roland, G. Roland, G. S. F. Stephans, K. Sumorok, D. Velicanu, J. Veverka, B. Wyslouch, M. Yang, M. Zanetti, V. Zhukova, B. Dahmes, A. Gude, S. C. Kao, K. Klapoetke, Y. Kubota, J. Mans, S. Nourbakhsh, R. Rusack, A. Singovsky, N. Tambe, J. Turkewitz, J. G. Acosta, S. Oliveros, E. Avdeeva, K. Bloom, S. Bose, D. R. Claes, A. Dominguez, R. Gonzalez Suarez, J. Keller, D. Knowlton, I. Kravchenko, J. Lazo-Flores, F. Meier, F. Ratnikov, G. R. Snow, M. Zvada, J. Dolen, A. Godshalk, I. Iashvili, A. Kharchilava, A. Kumar, S. Rappoccio, G. Alverson, E. Barberis, D. Baumgartel, M. Chasco, A. Massironi, D. M. Morse, D. Nash, T. Orimoto, D. Trocino, R. J. Wang, D. Wood, J. Zhang, K. A. Hahn, A. Kubik, N. Mucia, N. Odell, B. Pollack, A. Pozdnyakov, M. Schmitt, S. Stoynev, K. Sung, M. Velasco, S. Won, A. Brinkerhoff, K. M. Chan, A. Drozdetskiy, M. Hildreth, C. Jessop, D. J. Karmgard, N. Kellams, K. Lannon, S. Lynch, N. Marinelli, Y. Musienko, T. Pearson, M. Planer, R. Ruchti, G. Smith, N. Valls, M. Wayne, M. Wolf, A. Woodard, L. Antonelli, J. Brinson, B. Bylsma, L. S. Durkin, S. Flowers, A. Hart, C. Hill, R. Hughes, K. Kotov, T. Y. Ling, W. Luo, D. Puigh, M. Rodenburg, B. L. Winer, H. Wolfe, H. W. Wulsin, O. Driga, P. Elmer, J. Hardenbrook, P. Hebda, S. A. Koay, P. Lujan, D. Marlow, T. Medvedeva, M. Mooney, J. Olsen, P. Piroué, X. Quan, H. Saka, D. Stickland, C. Tully, J. S. Werner, A. Zuranski, E. Brownson, S. Malik, H. Mendez, J. E. Ramirez Vargas, V. E. Barnes, D. Benedetti, D. Bortoletto, M. De Mattia, L. Gutay, Z. Hu, M. K. Jha, M. Jones, K. Jung, M. Kress, N. Leonardo, D. H. Miller, N. Neumeister, F. Primavera, B. C. Radburn-Smith, X. Shi, I. Shipsey, D. Silvers, A. Svyatkovskiy, F. Wang, W. Xie, L. Xu, J. Zablocki, N. Parashar, J. Stupak, A. Adair, B. Akgun, K. M. Ecklund, F. J. M. Geurts, W. Li, B. Michlin, B. P. Padley, R. Redjimi, J. Roberts, J. Zabel, B. Betchart, A. Bodek, P. de Barbaro, R. Demina, Y. Eshaq, T. Ferbel, M. Galanti, A. Garcia-Bellido, P. Goldenzweig, J. Han, A. Harel, O. Hindrichs, A. Khukhunaishvili, S. Korjenevski, G. Petrillo, M. Verzetti, D. Vishnevskiy, R. Ciesielski, L. Demortier, K. Goulianos, C. Mesropian, S. Arora, A. Barker, J. P. Chou, C. Contreras-Campana, E. Contreras-Campana, D. Duggan, D. Ferencek, Y. Gershtein, R. Gray, E. Halkiadakis, D. Hidas, S. Kaplan, A. Lath, S. Panwalkar, M. Park, S. Salur, S. Schnetzer, D. Sheffield, S. Somalwar, R. Stone, S. Thomas, P. Thomassen, M. Walker, K. Rose, S. Spanier, A. York, O. Bouhali, A. Castaneda Hernandez, S. Dildick, R. Eusebi, W. Flanagan, J. Gilmore, T. Kamon, V. Khotilovich, V. Krutelyov, R. Montalvo, I. Osipenkov, Y. Pakhotin, R. Patel, A. Perloff, J. Roe, A. Rose, A. Safonov, I. Suarez, A. Tatarinov, K. A. Ulmer, N. Akchurin, C. Cowden, J. Damgov, C. Dragoiu, P. R. Dudero, J. Faulkner, K. Kovitanggoon, S. Kunori, S. W. Lee, T. Libeiro, I. Volobouev, E. Appelt, A. G. Delannoy, S. Greene, A. Gurrola, W. Johns, C. Maguire, Y. Mao, A. Melo, M. Sharma, P. Sheldon, B. Snook, S. Tuo, J. Velkovska, M. W. Arenton, S. Boutle, B. Cox, B. Francis, J. Goodell, R. Hirosky, A. Ledovskoy, H. Li, C. Lin, C. Neu, E. Wolfe, J. Wood, C. Clarke, R. Harr, P. E. Karchin, C. Kottachchi Kankanamge Don, P. Lamichhane, J. Sturdy, D. A. Belknap, D. Carlsmith, M. Cepeda, S. Dasu, L. Dodd, S. Duric, E. Friis, R. Hall-Wilton, M. Herndon, A. Hervé, P. Klabbers, A. Lanaro, C. Lazaridis, A. Levine, R. Loveless, A. Mohapatra, I. Ojalvo, T. Perry, G. A. Pierro, G. Polese, I. Ross, T. Sarangi, A. Savin, W. H. Smith, D. Taylor, C. Vuosalo, N. Woods, CMS Collaboration

**Affiliations:** Yerevan Physics Institute, Yerevan, Armenia; Institut für Hochenergiephysik der OeAW, Vienna, Austria; National Centre for Particle and High Energy Physics, Minsk, Belarus; Universiteit Antwerpen, Antwerpen, Belgium; Vrije Universiteit Brussel, Brussels, Belgium; Université Libre de Bruxelles, Brussels, Belgium; Ghent University, Ghent, Belgium; Université Catholique de Louvain, Louvain-la-Neuve, Belgium; Université de Mons, Mons, Belgium; Centro Brasileiro de Pesquisas Fisicas, Rio de Janeiro, Brazil; Universidade do Estado do Rio de Janeiro, Rio de Janeiro, Brazil; Universidade Estadual Paulista, Universidade Federal do ABC, São Paulo, Brazil; Institute for Nuclear Research and Nuclear Energy, Sofia, Bulgaria; University of Sofia, Sofia, Bulgaria; Institute of High Energy Physics, Beijing, China; State Key Laboratory of Nuclear Physics and Technology, Peking University, Beijing, China; Universidad de Los Andes, Bogotá, Colombia; Faculty of Electrical Engineering, Mechanical Engineering and Naval Architecture, University of Split, Split, Croatia; Faculty of Science, University of Split, Split, Croatia; Institute Rudjer Boskovic, Zagreb, Croatia; University of Cyprus, Nicosia, Cyprus; Charles University, Prague, Czech Republic; Academy of Scientific Research and Technology of the Arab Republic of Egypt, Egyptian Network of High Energy Physics, Cairo, Egypt; National Institute of Chemical Physics and Biophysics, Tallinn, Estonia; Department of Physics, University of Helsinki, Helsinki, Finland; Helsinki Institute of Physics, Helsinki, Finland; Lappeenranta University of Technology, Lappeenranta, Finland; DSM/IRFU, CEA/Saclay, Gif-sur-Yvette, France; Laboratoire Leprince-Ringuet, Ecole Polytechnique, IN2P3-CNRS, Palaiseau, France; Institut Pluridisciplinaire Hubert Curien, Université de Strasbourg, Université de Haute Alsace Mulhouse, CNRS/IN2P3, Strasbourg, France; Centre de Calcul de l’Institut National de Physique Nucleaire et de Physique des Particules, CNRS/IN2P3, Villeurbanne, France; Institut de Physique Nucléaire de Lyon, Université de Lyon, Université Claude Bernard Lyon 1, CNRS-IN2P3, Villeurbanne, France; Institute of High Energy Physics and Informatization, Tbilisi State University, Tbilisi, Georgia; I. Physikalisches Institut, RWTH Aachen University, Aachen, Germany; III. Physikalisches Institut A, RWTH Aachen University, Aachen, Germany; III. Physikalisches Institut B, RWTH Aachen University, Aachen, Germany; Deutsches Elektronen-Synchrotron, Hamburg, Germany; University of Hamburg, Hamburg, Germany; Institut für Experimentelle Kernphysik, Karlsruhe, Germany; Institute of Nuclear and Particle Physics (INPP), NCSR Demokritos, Aghia Paraskevi, Greece; University of Athens, Athens, Greece; University of Ioánnina, Ioannina, Greece; Wigner Research Centre for Physics, Budapest, Hungary; Institute of Nuclear Research ATOMKI, Debrecen, Hungary; University of Debrecen, Debrecen, Hungary; National Institute of Science Education and Research, Bhubaneswar, India; Panjab University, Chandigarh, India; University of Delhi, Delhi, India; Saha Institute of Nuclear Physics, Kolkata, India; Bhabha Atomic Research Centre, Mumbai, India; Tata Institute of Fundamental Research, Mumbai, India; Institute for Research in Fundamental Sciences (IPM), Tehran, Iran; University College Dublin, Dublin, Ireland; INFN Sezione di Bari, Università di Bari, Politecnico di Bari, Bari, Italy; INFN Sezione di Bologna, Università di Bologna, Bologna, Italy; INFN Sezione di Catania, Università di Catania, CSFNSM, Catania, Italy; INFN Sezione di Firenze, Università di Firenze, Florence, Italy; INFN Laboratori Nazionali di Frascati, Frascati, Italy; INFN Sezione di Genova, Università di Genova, Genoa, Italy; INFN Sezione di Milano-Bicocca, Università di Milano-Bicocca, Milan, Italy; INFN Sezione di Napoli, Università di Napoli ‘Federico II’, Naples, Italy, Università della Basilicata, Potenza, Italy, Università G. Marconi, Rome, Italy; INFN Sezione di Padova, Università di Padova, Padua, Italy, Università di Trento, Trento, Italy; INFN Sezione di Pavia, Università di Pavia, Pavia, Italy; INFN Sezione di Perugia, Università di Perugia, Perugia, Italy; INFN Sezione di Pisa, Università di Pisa, Scuola Normale Superiore di Pisa, Pisa, Italy; INFN Sezione di Roma, Università di Roma, Rome, Italy; INFN Sezione di Torino, Università di Torino, Turin, Italy, Università del Piemonte Orientale, Novara, Italy; INFN Sezione di Trieste, Università di Trieste, Trieste, Italy; Kangwon National University, Chunchon, Korea; Kyungpook National University, Daegu, Korea; Chonbuk National University, Jeonju, Korea; Chonnam National University, Institute for Universe and Elementary Particles, Kwangju, Korea; Korea University, Seoul, Korea; Seoul National University, Seoul, Korea; University of Seoul, Seoul, Korea; Sungkyunkwan University, Suwon, Korea; Vilnius University, Vilnius, Lithuania; National Centre for Particle Physics, Universiti Malaya, Kuala Lumpur, Malaysia; Centro de Investigacion y de Estudios Avanzados del IPN, Mexico City, Mexico; Universidad Iberoamericana, Mexico City, Mexico; Benemerita Universidad Autonoma de Puebla, Puebla, Mexico; Universidad Autónoma de San Luis Potosí, San Luis Potosí, Mexico; University of Auckland, Auckland, New Zealand; University of Canterbury, Christchurch, New Zealand; National Centre for Physics, Quaid-I-Azam University, Islamabad, Pakistan; National Centre for Nuclear Research, Swierk, Poland; Institute of Experimental Physics, Faculty of Physics, University of Warsaw, Warsaw, Poland; Laboratório de Instrumentação e Física Experimental de Partículas, Lisbon, Portugal; Joint Institute for Nuclear Research, Dubna, Russia; Petersburg Nuclear Physics Institute, Gatchina, (St. Petersburg), Russia; Institute for Nuclear Research, Moscow, Russia; Institute for Theoretical and Experimental Physics, Moscow, Russia; P. N. Lebedev Physical Institute, Moscow, Russia; Skobeltsyn Institute of Nuclear Physics, Lomonosov Moscow State University, Moscow, Russia; State Research Center of Russian Federation, Institute for High Energy Physics, Protvino, Russia; Faculty of Physics and Vinca Institute of Nuclear Sciences, University of Belgrade, Belgrade, Serbia; Centro de Investigaciones Energéticas Medioambientales y Tecnológicas (CIEMAT), Madrid, Spain; Universidad Autónoma de Madrid, Madrid, Spain; Universidad de Oviedo, Oviedo, Spain; Instituto de Física de Cantabria (IFCA), CSIC-Universidad de Cantabria, Santander, Spain; CERN, European Organization for Nuclear Research, Geneva, Switzerland; Paul Scherrer Institut, Villigen, Switzerland; Institute for Particle Physics, ETH Zurich, Zurich, Switzerland; Universität Zürich, Zurich, Switzerland; National Central University, Chung-Li, Taiwan; National Taiwan University (NTU), Taipei, Taiwan; Department of Physics, Faculty of Science, Chulalongkorn University, Bangkok, Thailand; Cukurova University, Adana, Turkey; Physics Department, Middle East Technical University, Ankara, Turkey; Bogazici University, Istanbul, Turkey; Istanbul Technical University, Istanbul, Turkey; National Scientific Center, Kharkov Institute of Physics and Technology, Kharkov, Ukraine; University of Bristol, Bristol, UK; Rutherford Appleton Laboratory, Didcot, UK; Imperial College, London, UK; Brunel University, Uxbridge, UK; Baylor University, Waco, USA; The University of Alabama, Tuscaloosa, USA; Boston University, Boston, USA; Brown University, Providence, USA; University of California, Davis, Davis, USA; University of California, Los Angeles, USA; University of California, Riverside, Riverside, USA; University of California, San Diego, La Jolla, USA; University of California, Santa Barbara, Santa Barbara, USA; California Institute of Technology, Pasadena, USA; Carnegie Mellon University, Pittsburgh, USA; University of Colorado at Boulder, Boulder, USA; Cornell University, Ithaca, USA; Fairfield University, Fairfield, USA; Fermi National Accelerator Laboratory, Batavia, USA; University of Florida, Gainesville, USA; Florida International University, Miami, USA; Florida State University, Tallahassee, USA; Florida Institute of Technology, Melbourne, USA; University of Illinois at Chicago (UIC), Chicago, USA; The University of Iowa, Iowa City, USA; Johns Hopkins University, Baltimore, USA; The University of Kansas, Lawrence, USA; Kansas State University, Manhattan, USA; Lawrence Livermore National Laboratory, Livermore, USA; University of Maryland, College Park, USA; Massachusetts Institute of Technology, Cambridge, USA; University of Minnesota, Minneapolis, USA; University of Mississippi, Oxford, USA; University of Nebraska-Lincoln, Lincoln, USA; State University of New York at Buffalo, Buffalo, USA; Northeastern University, Boston, USA; Northwestern University, Evanston, USA; University of Notre Dame, Notre Dame, USA; The Ohio State University, Columbus, USA; Princeton University, Princeton, USA; University of Puerto Rico, Mayagüez, USA; Purdue University, West Lafayette, USA; Purdue University Calumet, Hammond, USA; Rice University, Houston, USA; University of Rochester, Rochester, USA; The Rockefeller University, New York, USA; Rutgers, The State University of New Jersey, Piscataway, USA; University of Tennessee, Knoxville, USA; Texas A&M University, College Station, USA; Texas Tech University, Lubbock, USA; Vanderbilt University, Nashville, USA; University of Virginia, Charlottesville, USA; Wayne State University, Detroit, USA; University of Wisconsin, Madison, USA; CERN, Geneva, Switzerland

## Abstract

Measurements of the $$\mathrm{Z}$$$$\mathrm{Z}$$ production cross sections in proton–proton collisions at center-of-mass energies of 7 and 8$$~\mathrm{TeV}$$ are presented. Candidate events for the leptonic decay mode $$\mathrm{Z}\mathrm{Z}\rightarrow 2\mathrm{l} 2\nu $$, where $$\mathrm{l}$$ denotes an electron or a muon, are reconstructed and selected from data corresponding to an integrated luminosity of 5.1 (19.6)$$\,\text {fb}^\text {-1}$$ at 7 (8)$$~\mathrm{TeV}$$ collected with the CMS experiment. The measured cross sections, $$\sigma (\mathrm {p}\mathrm {p}\rightarrow \mathrm{Z}\mathrm{Z}) = 5.1_{-1.4}^{+1.5}\,\text {(stat)} \,_{-1.1}^{+1.4}\,\text {(syst)} \pm 0.1\,\text {(lumi)} \text {~pb} $$ at 7$$~\mathrm{TeV}$$, and $$7.2_{-0.8}^{+0.8}\,\text {(stat)} \,_{-1.5}^{+1.9}\,\text {(syst)} \,\pm \, 0.2\,\text {(lumi)} \text {~pb} $$ at 8$$~\mathrm{TeV}$$, are in good agreement with the standard model predictions with next-to-leading-order accuracy. The selected data are analyzed to search for anomalous triple gauge couplings involving the $$\mathrm{Z}$$$$\mathrm{Z}$$ final state. In the absence of any deviation from the standard model predictions, limits are set on the relevant parameters. These limits are then combined with the previously published CMS results for $$\mathrm{Z}$$$$\mathrm{Z}$$ in 4$$\mathrm{l}$$ final states, yielding the most stringent constraints on the anomalous couplings.

## Introduction

The production of pairs of $$\mathrm{Z}$$ bosons in proton–proton collisions is a rare diboson process in the Standard Model (SM). The measurement of the cross section and properties of this process probe the self-interaction of electroweak gauge bosons. The $$\mathrm{Z}$$$$\mathrm{Z}$$ final state is also an important background in searches for other interesting processes beyond the SM, such as the production of high-mass Higgs bosons and their subsequent decay to pairs of bosons [1] or supersymmetry [2]. Because of the non-Abelian structure of the electroweak gauge theory, vector bosons can interact among themselves and can couple in triplets (e.g. $$\mathrm {W}$$$$\mathrm {W}$$$$\mathrm{Z}$$) or quartets (e.g. $$\mathrm {W}$$$$\mathrm {W}$$$$\mathrm{Z}$$$$\mathrm{Z}$$). All couplings involving only bosons without electric charge are expected to be null at tree level, leading to the absence of triple gauge couplings for $$\mathrm{Z}$$$$\gamma $$$$\gamma $$, $$\mathrm{Z}$$$$\mathrm{Z}$$$$\gamma $$, and $$\mathrm{Z}$$$$\mathrm{Z}$$$$\mathrm{Z}$$. An enhancement in the measured rate of $$\mathrm{Z}$$$$\mathrm{Z}$$ production compared to the expectation from the SM could indicate the existence of anomalous boson couplings.

This paper presents measurements of the $$\mathrm{Z}$$$$\mathrm{Z}$$ production cross sections in proton–proton collisions at the LHC at two different center-of-mass energies, 7 and 8$$~\mathrm{TeV}$$, in the decay channel with two charged leptons, electrons ($$\mathrm {e}$$$$\mathrm {e}$$) or muons ($$\mathrm {\mu }$$$$\mathrm {\mu }$$), and a neutrino-antineutrino pair of any flavor ($$\nu \bar{\nu }$$). The data were collected with the CMS detector at 7 (8)$$~\mathrm{TeV}$$, corresponding to 5.1 (19.6)$$\,\text {fb}^\text {-1}$$ of integrated luminosity.

At tree level, $$\mathrm{Z}$$$$\mathrm{Z}$$ pairs are primarily produced in the SM via the *t*- and *u*-channels, following the annihilation of a quark–antiquark pair in proton–proton collisions. Because of the high gluon–gluon parton luminosity, the $$\mathrm{g}\mathrm{g}\rightarrow \mathrm{Z}\mathrm{Z}$$ contribution has to be included. The production cross section calculated up to next-to-leading-order (NLO) accuracy in strong coupling constant ($$\alpha _\mathrm {S}$$) is expected to be $$6.46^{+0.30}_{-0.21}$$ ($$7.92^{+0.37}_{-0.24}$$)$$\text {~pb}$$ at 7 (8)$$~\mathrm{TeV}$$  [3], where the uncertainties refer only to the missing higher orders in the computation. These cross sections include a leading-order (LO) computation of the $$\mathrm{g}\mathrm{g}\rightarrow \mathrm{Z}\mathrm{Z}$$ contribution, which is formally a next-to-next-to-leading-order (NNLO) correction. Recently, complete NNLO cross sections for $$\mathrm{Z}$$$$\mathrm{Z}$$ production accompanied by jets have also been computed [4], leading to a further small increase in cross section compared to Ref. [3]. However, higher-order QCD corrections have been shown to be reduced significantly when vetoing events where the diboson system is produced in association with jets [5, 6], as done in the present analysis. The NNLO QCD corrections apart from the LO $$\mathrm{g}\mathrm{g}\rightarrow \mathrm{Z}\mathrm{Z}$$ contribution are thus neglected in our simulations and in the reference cross sections to which our measurements are compared. Complete one-loop electroweak (EW) corrections to massive vector boson pair production [7, 8] have also been published. The consequences of the EW corrections for $$\mathrm{Z}$$$$\mathrm{Z}$$ production are that the transverse momentum ($$p_{\mathrm {T}}$$) spectrum of the $$\mathrm{Z}$$ bosons falls more rapidly and, in addition, the overall cross section decreases by about 4 % at LHC center-of-mass energies.

The production of $$\mathrm{Z}$$$$\mathrm{Z}$$ pairs has been studied at the LHC by the ATLAS experiment, which analyzed the decay modes $$2\mathrm{l} 2\mathrm{l} ^{\prime }$$ and $$2\mathrm{l} 2\nu $$ ($$\mathrm{l},\mathrm{l} ^{\prime }=\mathrm {e},\mathrm {\mu }$$) at 7$$~\mathrm{TeV}$$  [9], and by the CMS experiment, which considered $$2\mathrm{l} 2\mathrm{l} ^{\prime }$$ final states ($$\mathrm{l} =\mathrm {e},\mathrm {\mu }$$ and $$\mathrm{l} ^{\prime }=\mathrm {e},\mathrm {\mu },\mathrm {\tau }$$) at 7$$~\mathrm{TeV}$$  [10] and 8$$~\mathrm{TeV}$$  [11]. Both experiments measured $$\mathrm{Z}$$$$\mathrm{Z}$$ production cross sections in good agreement with the SM predictions and set limits on anomalous triple gauge couplings (ATGCs).

The branching fraction for the $$2\mathrm{l} 2\nu $$ decay mode (where $$\mathrm{l}$$ denotes only $$\mathrm {e}$$ and $$\mathrm {\mu }$$) is approximately six times larger than that of the four-charged-lepton final state, and the signal purity is enhanced at large values of the boson $$p_{\mathrm {T}}$$, where there is the greatest sensitivity to ATGC effects. For this reason, the $$2\mathrm{l} 2\nu $$ channel has a sensitivity comparable to that of the 4$$\mathrm{l}$$ channel to ATGC. The characteristic signature is an overall imbalance in the transverse momentum of the event between the initial and the final states, which consequently appears as missing transverse energy ($$E_{\mathrm{T}}^{\mathrm{miss}}$$) in the final state. Although the branching fraction is large, this channel is rather challenging due to the large contamination from background processes, in particular the Drell–Yan (DY) process, which has a cross section nearly five orders of magnitude larger than the signal. If the $$\mathrm{Z}$$ boson or the hadrons recoiling against it are not reconstructed correctly, then an apparent $$E_{\mathrm{T}}^{\mathrm{miss}}$$ results and these events can resemble the signal. Other important sources of background are diboson processes, $$\mathrm {W}$$$$\mathrm {W}$$ and $$\mathrm {W}$$$$\mathrm{Z}$$, with fully leptonic decays, and $$\mathrm{t} \bar{\mathrm{t}}$$ production.

This paper presents a measurement of the $$\mathrm{Z}$$$$\mathrm{Z}$$ production cross section in the $$2\mathrm{l} 2\nu $$ channel as a function of the transverse momentum ($$p_{\mathrm {T}}$$) of the charged lepton pair. The distribution of the dilepton $$p_{\mathrm {T}}$$ is sensitive to the presence of ATGCs. Limits are computed and finally combined with existing results from CMS in the four-charged-lepton final state.

## CMS detector

The central feature of the CMS apparatus is a superconducting solenoid of 6$\text {~m}$ internal diameter, providing a magnetic field of 3.8$\text {~T}$. Within the superconducting solenoid volume are a silicon pixel and strip tracker, a lead tungstate crystal electromagnetic calorimeter (ECAL), and a brass and scintillator hadron calorimeter (HCAL), each composed of a barrel and two endcap sections. The silicon tracking system is used to measure the momentum of charged particles and covers the pseudorapidity range $|\eta | < 2.5$, where $\eta = -\ln {\left( \tan {\left( \theta /2\right) }\right) }$, and $\theta$ is the polar angle of the trajectory of the particle with respect to the counterclockwise-beam direction. The ECAL and HCAL extend to a pseudorapidity range of $|\eta | < 3.0$. A steel/quartz-fiber Cherenkov forward detector extends the calorimetric coverage to $|\eta | < 5.0$. Muons are measured in gas-ionization detectors embedded in the steel flux-return yoke outside the solenoid.

The $E_{\mathrm{T}}^{\mathrm{miss}}$ is defined as the magnitude of the missing transverse momentum or momentum imbalance, $\mathbf {p_{\mathrm {T}} ^{\text {miss}}}$, which is the negative vector sum of the momenta in the plane transverse to the beam of all reconstructed particles (photons, electrons, muons, charged and neutral hadrons) in the event.

A more detailed description of the CMS detector, together with a definition of the coordinate system used and the relevant kinematic variables, can be found in Ref. [12].

## Simulation

Several Monte Carlo (MC) event generators are used to simulate the signal and background processes. The $\mathrm{Z}\mathrm{Z}\rightarrow 2\mathrm{l} 2\nu$ signal and the $\mathrm {W}\mathrm {W}\rightarrow 2\mathrm{l} 2\nu$ and $\mathrm {W}\mathrm{Z}\rightarrow 3\mathrm{l} \nu$ background processes are simulated using MadGraph 5 [13], as well as $\mathrm{Z}$ $+$ jets, $\mathrm {W}$ $+$ jets, and $\mathrm{t} \bar{\mathrm{t}}$ $+$ jets processes. Single top-quark processes are simulated with powheg  [14]. In the simulation, vector bosons are allowed to decay to leptons of any flavor ($\mathrm {e}$, $\mathrm {\mu }$, $\mathrm {\tau }$), since $\mathrm {\tau }$ leptons can contribute to dielectron and dimuon final states through $\mathrm {\tau }\rightarrow \mathrm {e}$ and $\mathrm {\tau }\rightarrow \mathrm {\mu }$ decays. For all these processes, the parton showering is simulated with pythia 6 [15] with the Z2 (Z2*) tune for 7 (8)$~\mathrm{TeV}$ simulations [16].

The cross section of the $$\mathrm{Z}$$$$\mathrm{Z}$$ signal is computed with the NLO generator mcfm  [3], which includes contributions from gluon–gluon initial states. Since the present cross section measurement and ATGC analysis rely on the $$p_{\mathrm {T}}$$ distribution of $$\mathrm{Z}$$ bosons, a precise prediction of this distribution is required. The charged dilepton $$p_{\mathrm {T}}$$ spectrum of $$\mathrm{Z}\mathrm{Z}\rightarrow 2\mathrm{l} 2\nu $$, generated with MadGraph and interfaced with pythia for parton showering, is found to be in good agreement with the corresponding spectrum computed at NLO in QCD with mcfm and therefore no differential correction for NLO QCD effects is applied to the MadGraph simulated sample. In addition, the effect of NLO EW corrections [7, 8] is taken into account by reweighting the $$\mathrm{Z}$$$$\mathrm{Z}$$ and the $$\mathrm {W}$$$$\mathrm{Z}$$ events as a function of the partonic kinematic variables, and applying weights derived from the calculations described in Ref. [7]. These corrections yield an overall reduction of $$4.1\,\%$$ of the $$\mathrm{Z}$$$$\mathrm{Z}$$ cross section, as well as a softening of the boson $$p_{\mathrm {T}}$$ spectra that results in a reduction of the differential cross section of about 20 % at $$\mathrm{Z}$$$$p_{\mathrm {T}}$$ of 300$$\,\text {GeV}$$.

Simulated samples of the $\mathrm{Z}\mathrm{Z}\rightarrow 2\mathrm{l} 2\nu$ process that include contributions from ATGCs (see Sect. 8) are produced using the LO generator sherpa  [17]. These samples are based on a LO matrix-element simulation including up to two additional jets, matched to parton showers.

The parton distribution functions (PDF) are modeled with the CTEQ6L [18] parametrization in samples generated with MadGraph, and the CT10 parametrization [19] in samples generated with powheg and sherpa. The detector response to the simulated events is modeled with Geant4  [12, 20].

## Event selection

The signal consists of two $\mathrm{Z}$ bosons, one decaying into a pair of oppositely charged leptons and the other to two neutrinos that escape direct detection. The final state is thus characterized by: a pair of oppositely charged, isolated electrons or muons, with an invariant mass within a $\mathrm{Z}$-boson mass window, no additional leptons, and large $E_{\mathrm{T}}^{\mathrm{miss}}$.

Events are selected using triggers that require the presence of two electrons or two muons, with minimum $p_{\mathrm {T}}$ thresholds on each lepton that depend on the dataset. The trigger thresholds in the 8$~\mathrm{TeV}$ dataset are 17 and 8$\,\text {GeV}$ for the leptons with higher and lower $p_{\mathrm {T}}$, respectively. The thresholds for the 7$~\mathrm{TeV}$ data samples are the same or lower. The 8$~\mathrm{TeV}$ data sample also includes events that satisfy a single isolated muon trigger to ensure the highest efficiency. For events with two identified and isolated leptons having invariant mass between 83.5 and 98.5$\,\text {GeV}$ and dilepton $p_{\mathrm {T}} >45\,\text {GeV}$, the trigger efficiency is higher than 98 % in the dielectron channel and varies from 94 to 98 % in the dimuon channel. In addition, single-photon triggers or electron-muon triggers are used to select control samples for the background determinations.

Electrons are selected inside the fiducial region of ECAL. The electron candidates must have a minimum $p_{\mathrm {T}}$ of 20$\,\text {GeV}$, and satisfy standard identification criteria, based on shower shape, track quality, cluster track matching, in order to reject misidentified hadrons [21].

The muons are selected inside the fiducial region of the muon spectrometer, with a minimum $p_{\mathrm {T}}$ of 20$\,\text {GeV}$, and satisfy standard identification criteria based on track information and isolation [22].

**Table 1:** Summary of the optimal signal selection

Variable	Value
Dilepton invariant mass	$|m(\mathrm{l} \mathrm{l})-91 | < 7.5$ $\,\text {GeV}$
Dilepton $p_{\mathrm {T}}$	$p_{\mathrm {T}} ^{\mathrm{l} \mathrm{l}} > 45$ $\,\text {GeV}$
$\mathrm{b}$-tagged jets	Based on vertex info (for jet with $p_{\mathrm {T}} > 20$ $\,\text {GeV}$)
Jet veto	No jets with $p_{\mathrm {T}} > 30\,\text {GeV}$
Reduced $E_{\mathrm{T}}^{\mathrm{miss}}$	$>$65$\,\text {GeV}$
$E_{\mathrm{T}}^{\mathrm{miss}}$ balance	$0.4 < E_{\mathrm{T}}^{\mathrm{miss}}/p_{\mathrm {T}} ^{\mathrm{l} \mathrm{l}} < 1.8$
$\Delta \phi$($\mathbf {p_{\mathrm {T}} ^{\text {miss}}}$, jet)	$>$0.5 rad
$\Delta \phi$($\mathbf {p_{\mathrm {T}} ^{\text {miss}}}$, lepton)	$>$0.2 rad
Lepton veto	No additional leptons ($\mathrm {e}$/$\mathrm {\mu }$) with $p_{\mathrm {T}} >10/3\,\text {GeV}$

Events are selected if they include a pair of same-flavor, oppositely charged leptons that pass the identification and isolation criteria. In order to suppress backgrounds that do not include a $$\mathrm{Z}$$ boson, the lepton pair is required to have an invariant mass compatible with the $$\mathrm{Z}$$-boson mass, between 83.5 and 98.5$$\,\text {GeV}$$. The $$p_{\mathrm {T}}$$ of the dilepton pair is required to be greater than 45$$\,\text {GeV}$$. This requirement is particularly effective at reducing the DY background because the $$\mathrm{Z}$$ bosons produced in $$\mathrm{Z}$$$$\mathrm{Z}$$ events have, on average, larger $$p_{\mathrm {T}}$$ than those from single $$\mathrm{Z}$$-boson production.

Since the $$\mathrm{Z}$$$$\mathrm{Z}$$ pair is produced in the collision of two hadrons, the event might have jets from initial-state radiation. We use jets reconstructed from particle-flow (PF) candidates, using the anti-$$k_\mathrm{T}$$ algorithm [23] with a distance parameter of 0.5. The jet transverse energy is corrected using the CMS standard prescriptions for jet energy scale (JES) calibration [24]. Only jets with a corrected $$p_{\mathrm {T}}$$ greater than 10$$\,\text {GeV}$$ and reconstructed within $$|\eta |<5$$ are used in this analysis. Further corrections are applied to reduce the effect of secondary proton–proton collisions overlapping with the primary interaction (pileup). An extra correction is applied to jets in the MC samples to match the resolution observed in data. In order to reject jets dominated by instrumental and beam-related noise, loose identification criteria are applied, based on the multiplicity and energy fraction of charged and neutral particles.

In order to suppress background coming from top quarks, events are vetoed if they have a jet identified as a $\mathrm{b}$-quark jet ($\mathrm{b}$-tagged). A requirement based on a combined secondary vertex discriminator [25] is applied to $\mathrm{b}$-tagged jets with $p_{\mathrm {T}} >20\,\text {GeV}$ within the tracker fiducial region ($|\eta |<2.4$). The misidentification probability for light-parton jets is about 10 %, whereas the efficiency for $\mathrm{b}$-jets is more than 80 %. To further reduce top-quark and other backgrounds with hadronic activity, events are rejected if they contain any jet with $p_{\mathrm {T}} >30\,\text {GeV}$.

A good $E_{\mathrm{T}}^{\mathrm{miss}}$ measurement is critical for the extraction of the $\mathrm{Z}\mathrm{Z}\rightarrow 2\mathrm{l} 2\nu$ signal given that the $E_{\mathrm{T}}^{\mathrm{miss}}$ distinguishes this process from the DY background. Since the average $E_{\mathrm{T}}^{\mathrm{miss}}$ of the signal is moderate ($\sim$50$\,\text {GeV}$), we cannot simply require a high $E_{\mathrm{T}}^{\mathrm{miss}}$. We follow the approach of constructing a “reduced $E_{\mathrm{T}}^{\mathrm{miss}}$ ” variable, as done in the D0 [26, 27] and OPAL [28] experiments. The concept behind a reduced $E_{\mathrm{T}}^{\mathrm{miss}}$ is to reduce the instrumental contribution to mismeasured $E_{\mathrm{T}}^{\mathrm{miss}}$ by considering possible contributions to fake $E_{\mathrm{T}}^{\mathrm{miss}}$. In each event, $\mathbf {p_{\mathrm {T}} ^{\text {miss}}}$ and jet momenta are decomposed along an orthogonal set of axes in the transverse plane of the detector. One of the axes is defined by the $\mathbf {p_{\mathrm {T}}}$ of the charged dilepton system, the other perpendicular to it. We define the recoil of the $\mathrm{l} ^+\mathrm{l} ^-$ system in two different ways: (1) the clustered recoil ($\mathbf {R_{\mathrm {c}}}$) is the vectorial sum of the momenta of the PF jets reconstructed in the event, and (2) the unclustered recoil ($\mathbf {R_{\mathrm {u}}}$) is the vectorial sum of the transverse momenta of all PF candidates in the event, with the exception of the two leptons. On each axis ($i=$ parallel/orthogonal to the dilepton system $\mathbf {p_{\mathrm {T}}}$), the reduced $E_{\mathrm{T}}^{\mathrm{miss}}$ projection is defined as

$$\begin{aligned} \text {reduced } {E_{\mathrm{T}}^{\mathrm{miss}}}^{i} = -p_{\mathrm {T}} ^{\mathrm{l} \mathrm{l},i} - R_{\mathrm {c/u}}^i\,, \end{aligned}$$

where $R_{\mathrm {c/u}}^i$ represents the choice of $R_{\mathrm {c}}$ or $R_{\mathrm {u}}$ that minimizes the absolute value of that reduced $E_{\mathrm{T}}^{\mathrm{miss}}$ component, and $p_{\mathrm {T}} ^{\mathrm{l} \mathrm{l},i}$ is a projection of the transverse momentum of the $\mathrm{Z}$ boson. The presence of genuine $E_{\mathrm{T}}^{\mathrm{miss}}$ in the recoil of the charged dilepton system is expected to be evident in the parallel projection, while the component perpendicular to the $\mathrm{l} ^{+}\mathrm{l} ^{-}$ system is mostly dominated by jet and $E_{\mathrm{T}}^{\mathrm{miss}}$ resolution. The absolute reduced $E_{\mathrm{T}}^{\mathrm{miss}}$ variable is the sum in quadrature of the two components. The reduced $E_{\mathrm{T}}^{\mathrm{miss}}$ shows better DY background suppression than the standard PF $E_{\mathrm{T}}^{\mathrm{miss}}$ at the same signal efficiency. It is also found to be more stable than the PF $E_{\mathrm{T}}^{\mathrm{miss}}$ under variations in pileup conditions and JES.

The $E_{\mathrm{T}}^{\mathrm{miss}}$ balance variable is defined as the ratio between the PF $E_{\mathrm{T}}^{\mathrm{miss}}$ and the transverse momentum of the leptonically decaying $\mathrm{Z}$ boson, namely $E_{\mathrm{T}}^{\mathrm{miss}}/p_{\mathrm {T}} ^{\mathrm{l} \mathrm{l}}$. Values of this variable far from unity identify events in which the leptonic $\mathrm{Z}$-boson candidate is not well balanced by genuine $E_{\mathrm{T}}^{\mathrm{miss}}$ from neutrinos, but recoils against mismeasured jets or leptons. The selected sample can still be contaminated by events with jets with $p_{\mathrm {T}}$ below the veto threshold.

A mismeasurement of the jet energy can produce mismeasured $\mathbf {p_{\mathrm {T}} ^{\text {miss}}}$ aligned with the jet direction in the transverse plane. These events are characterized by a small azimuthal angle between the $\mathbf {p_{\mathrm {T}} ^{\text {miss}}}$ vector and the closest jet, $\Delta \phi$($\mathbf {p_{\mathrm {T}} ^{\text {miss}}}$, jet). This distribution is used to reject $\mathrm{Z}$ $+$ jets events that have a small $\Delta \phi$ angle. The mismeasurement of a lepton $p_{\mathrm {T}}$ can also produce mismeasured $E_{\mathrm{T}}^{\mathrm{miss}}$. Although this effect is usually negligible, given the good lepton momentum resolution in CMS, events are found where a large $E_{\mathrm{T}}^{\mathrm{miss}}$ value ($>$60$\,\text {GeV}$) is accompanied by a small angle between the $\mathbf {p_{\mathrm {T}} ^{\text {miss}}}$ and the $\mathbf {p_{\mathrm {T}}}$ of a lepton. Events with $E_{\mathrm{T}}^{\mathrm{miss}} >60\,\text {GeV}$ and $\Delta \phi (\mathbf {p_{\mathrm {T}} ^{\text {miss}}}, \text {lepton})<$0.2 rad are therefore rejected.

In order to suppress the $$\mathrm {W}$$$$\mathrm{Z}$$ background, with both bosons decaying leptonically, events are required to have no additional leptons. To improve the rejection power, the $$p_{\mathrm {T}}$$ threshold is lowered to 3$$\,\text {GeV}$$ for additional muons, and 10$$\,\text {GeV}$$ for electrons. Furthermore, these muons and electrons are selected with looser criteria than those used to reconstruct the $$\mathrm{Z}$$-boson candidate.

The variables described above are used to extract the signal sample for the cross section measurement. We optimize the requirements in the final selection in order to minimize the total uncertainty in the measured cross section at 8$$~\mathrm{TeV}$$ (see Sect. 7). The same selection is applied to the 7$$~\mathrm{TeV}$$ data. For this purpose, we scan a series of possible analysis selections, in which we vary the dilepton mass window and $$p_{\mathrm {T}}$$ threshold, the minimum $$p_{\mathrm {T}}$$ of jets used in the computation of the reduced $$E_{\mathrm{T}}^{\mathrm{miss}}$$ variable, and the reduced $$E_{\mathrm{T}}^{\mathrm{miss}}$$ requirement. We optimize the selection using MC estimates of the background processes, or using predictions based on control samples in data from the DY, top-quark, and $$\mathrm {W}$$$$\mathrm {W}$$ backgrounds, as described in Sect. 5, and we find similar results for the optimal requirements and for the measured cross section. For the final optimization we choose the selection obtained using background estimates from data. The requirements are summarized in Table 1. With this selection, the acceptance for $$\mathrm{Z}\mathrm{Z}\rightarrow 2\mathrm {e}2\nu $$ and $$\mathrm{Z}\mathrm{Z}\rightarrow 2\mathrm {\mu }2\nu $$ events is about 10 % for both channels, at 7 and 8$$~\mathrm{TeV}$$.

## Background estimation

Although the DY process does not include genuine $E_{\mathrm{T}}^{\mathrm{miss}}$ from neutrinos, the tail of the reduced $E_{\mathrm{T}}^{\mathrm{miss}}$ distribution can be contaminated by these events due to detector energy resolution, jet energy mismeasurements, pileup energy fluctuations, and instrumental noise. Given that the simulation may not fully reproduce detector and pileup effects on the reduced $E_{\mathrm{T}}^{\mathrm{miss}}$ distribution, especially in the tails, and that the simulation is limited in statistical precision, we build a model of DY background from control samples in data. For this purpose we use a process that has similar jet multiplicity, underlying event, and pileup conditions as the DY process for the region of interest at high boson $p_{\mathrm {T}}$: the production of prompt isolated photons in association with jets ($\gamma$ + jets) [29]. We expect that an accurate description of the $E_{\mathrm{T}}^{\mathrm{miss}}$ distribution and other related kinematic variables can be obtained from this photon + jets sample. However, some corrections must be applied to the photon + jets sample to ensure a good modeling of the DY process. The yield of photon events is scaled to the observed charged dilepton system yield as a function of the boson $p_{\mathrm {T}}$ after applying the jet veto to both samples. This accounts for the differences in the selection efficiency of the dilepton and photon candidates and corrects for the trigger prescales, which are applied to the low-$p_{\mathrm {T}}$ photon triggers.

Only photons in the barrel region are used because the purity and resolution are better than in other regions. Following Ref. [1], the selection of photon events is based on shower shape, isolation in the tracker, and energy deposits in ECAL, and HCAL. After this selection, several processes with instrumental $$E_{\mathrm{T}}^{\mathrm{miss}}$$ contribute to the photon sample: single $$\gamma $$ events, double $$\gamma $$ events where one photon escapes detection or fails the identification, and QCD events with a jet misidentified as a photon. Processes with genuine $$E_{\mathrm{T}}^{\mathrm{miss}}$$ can also contaminate this sample: $$\mathrm {W}$$/$$\mathrm{Z}$$+$$\gamma $$ with the $$\mathrm {W}$$/$$\mathrm{Z}$$ boson decaying to $$\mathrm{l} \nu $$/$$\nu \nu $$, or $$\mathrm {W}$$ $$+$$ jets with the $$\mathrm {W}$$ boson decaying to $$\mathrm {e}$$$$\nu $$ and the electron misreconstructed as a photon. Although these processes have generally lower cross sections, they are characterized by large $$E_{\mathrm{T}}^{\mathrm{miss}}$$ values, and thus contribute to the tails of the distribution, where it is most important to measure the residual instrumental background. In order to reduce these background contributions, specific selections are applied. The event must have exactly one photon and no leptons. Only jets with $$\Delta R = \sqrt{{\left( \Delta \phi \right) ^2 + \left( \Delta \eta \right) ^2}} > 0.4$$ from the photon are used for all the jet-related selections (jet veto, reduced $$E_{\mathrm{T}}^{\mathrm{miss}}$$, etc.). To avoid misreconstruction of the photon energy, a conversion veto is applied using the number of missing expected tracker hits and the distance of closest approach between the reconstructed conversion tracks.

The remaining contribution from $\mathrm {W}+\gamma$ and $\mathrm {W}/\mathrm{Z}+\gamma$ events after this selection is estimated from simulation and subtracted from the photon data model. For this purpose, a set of simulated photon samples is used that includes $\gamma$ $+$ jets, QCD events with a jet misidentified as a photon (generated with pythia), $\mathrm {W}+\gamma \rightarrow \mathrm{l} \nu \gamma$, and $\mathrm{Z}+\gamma \rightarrow \nu \nu \gamma$ (generated with MadGraph). These samples are normalized to their respective cross sections computed at NLO in QCD. The full set of MC samples is reweighted and corrected following the same procedure as that used for the photon data sample. Finally, the photon data are corrected as a function of $E_{\mathrm{T}}^{\mathrm{miss}}$ by multiplying them by unity minus the fraction of electroweak processes in the simulation.

We apply a different data-based method to estimate the total number of background events from processes that do not involve a $$\mathrm{Z}$$ boson: i.e. $$\mathrm {W}$$$$\mathrm {W}$$ and top-quark production. We denote these events as nonresonant background (NRB). In order to measure this contribution, a control sample based on $$\mathrm {e}$$$$\mathrm {\mu }$$ candidate events is selected by applying the same requirements as in the main analysis. The NRB yields in the same-flavor channels ($$\mathrm {e}$$$$\mathrm {e}$$ and $$\mathrm {\mu }$$$$\mathrm {\mu }$$) are obtained by scaling the number of events in the control sample. The rescaling is done by means of correction factors, measured from the sidebands (SB) of the $$\mathrm{Z}$$-boson mass peak, i.e. in the regions 55–70 and 110–200$$\,\text {GeV}$$. The scale factors are measured in a looser selection region in order to improve the statistical precision. We require the reduced $$E_{\mathrm{T}}^{\mathrm{miss}} > 65$$$$\,\text {GeV}$$ in order to suppress the DY contribution from $$\mathrm {\tau }^+\mathrm {\tau }^-$$. We also require at least one $$\mathrm{b}$$-tagged jet with $$p_{\mathrm {T}} >20\,\text {GeV} $$, to further reduce DY and other backgrounds, and increase the fraction of top-quark events. The scale factors are defined as follows:

1 $$\begin{aligned} \alpha _{\mathrm {e}\mathrm {e}/\mathrm {\mu }\mathrm {\mu }} = N^\mathrm {SB}_{\mathrm {e}\mathrm {e}/\mathrm {\mu }\mathrm {\mu }} / N^\mathrm {SB}_{\mathrm {e}\mathrm {\mu }}, \end{aligned}$$

and the NRB contamination in the $\mathrm{Z}$-peak region is:

2 $$\begin{aligned} N^\text {peak}_{\mathrm {e}\mathrm {e}/\mathrm {\mu }\mathrm {\mu }} = \alpha _{\mathrm {e}\mathrm {e}/\mathrm {\mu }\mathrm {\mu }}\, N^\text {peak}_{\mathrm {e}\mathrm {\mu }}. \end{aligned}$$

The validity of the method is tested in simulation by comparing the predicted background to the expected number of $$\mathrm {W}$$$$\mathrm {W}$$ and top-quark events.

**Fig. 1:**
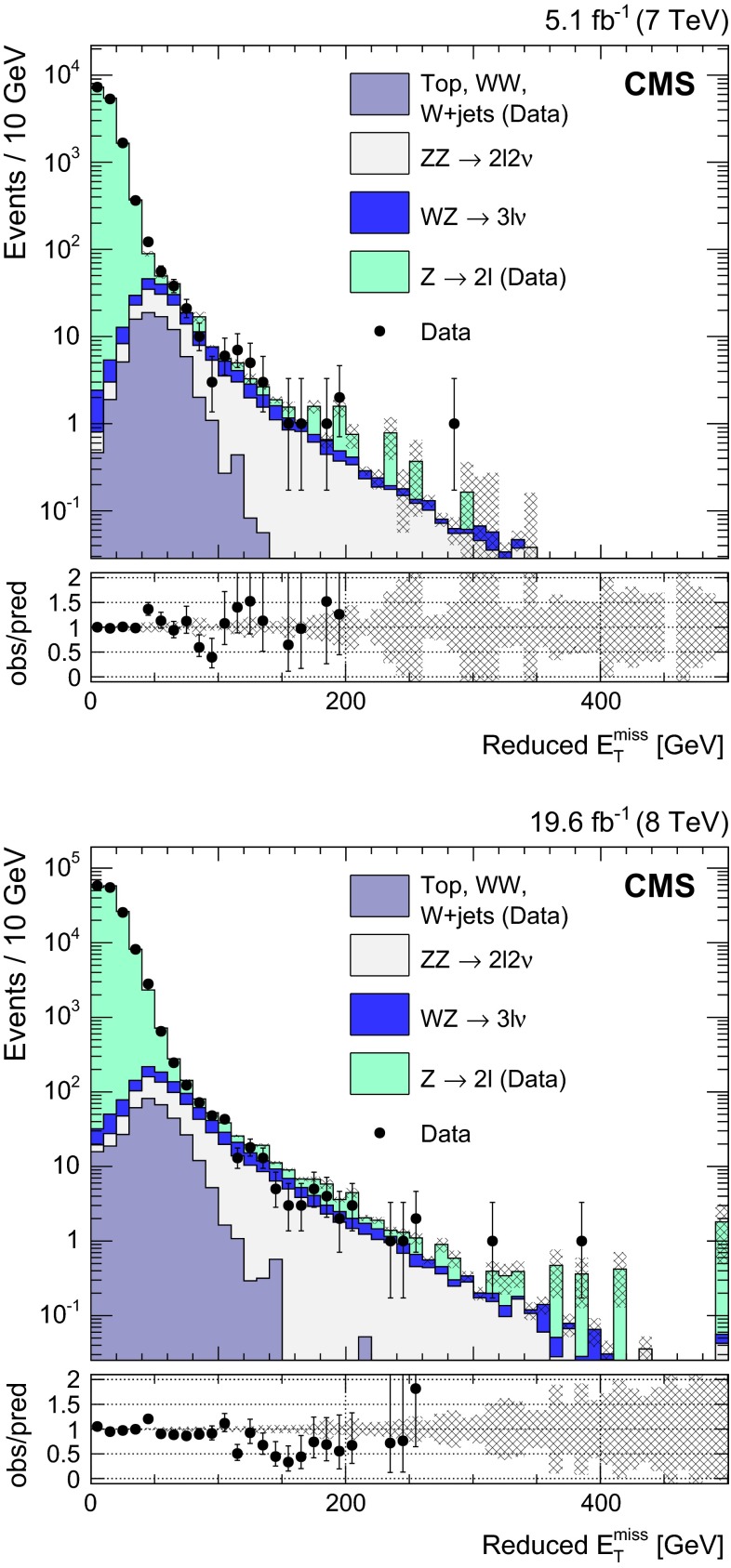
Reduced $E_{\mathrm{T}}^{\mathrm{miss}}$ spectrum in the inclusive $\mathrm{l} \mathrm{l}$ ($\mathrm{l} = \mathrm {e}, \mathrm {\mu }$) channel at 7$~\mathrm{TeV}$ (*top*) and 8$~\mathrm{TeV}$ (*bottom*), using the photon model to describe the DY contribution and NRB modeling for $\mathrm {W}$ $\mathrm {W}$, $\mathrm {W}$ + jets, and top-quark production, after selections on the dilepton invariant mass and $p_{\mathrm {T}}$, jet veto, $\mathrm{b}$-tagged jet veto, third lepton veto, and $\Delta \phi$($\mathbf {p_{\mathrm {T}} ^{\text {miss}}}$, jet), as described in Sect. 4. The *gray error band* represents the statistical uncertainty in the predicted yields

Figure 1 shows the reduced $E_{\mathrm{T}}^{\mathrm{miss}}$ distributions in dilepton data and simulation, using the photon model to describe the DY background and the data-driven estimation for NRB. A good agreement is found in the region dominated by the DY process, up to about 80$\,\text {GeV}$, while the higher part of the spectrum is dominated by diboson production. The error bands shown in Fig. 1 represent the statistical uncertainty in the predicted yields. A systematic uncertainty in the final DY event yield estimated with this method is computed as the relative difference between dilepton yields in data and simulation, in a control region with $E_{\mathrm{T}}^{\mathrm{miss}} <60\,\text {GeV}$, and it has been found to be 25 % (40 %) at 7 (8)$~\mathrm{TeV}$. This systematic uncertainty is not shown in Fig. 1.

## Systematic uncertainties

Different sources of systematic uncertainty are associated with the expected yields and distributions of signal and background processes and of the data. The uncertainties reported in the following paragraphs affect the final event yields of the relevant processes.

### Statistical uncertainty of the simulated and control samples

For the processes estimated from simulation, $$\mathrm{Z}$$$$\mathrm{Z}$$ and $$\mathrm {W}$$$$\mathrm{Z}$$, the limited size of the MC sample affects the precision of the modeling, and is therefore taken as a systematic uncertainty in the shape of the kinematic distributions used in the cross section measurement and ATGC limit setting. Similarly, the backgrounds estimated from data are limited by the size of the control samples described in Sect. 5: the $$\mathrm {e}$$$$\mathrm {\mu }$$ sample for nonresonant backgrounds and the $$\gamma $$ $$+$$ jets sample for DY background. These uncertainties are treated in the same way as those backgrounds that are estimated from simulation. This systematic uncertainty has been computed in different reduced $$E_{\mathrm{T}}^{\mathrm{miss}}$$ bins or different $$p_{\mathrm {T}}$$ bins and is used as shape errors in the fit.

### Cross sections of $$\mathrm{Z}$$$$\mathrm{Z}$$ and $$\mathrm {W}$$$$\mathrm{Z}$$

The cross sections for $$\mathrm {p}\mathrm {p}\rightarrow \mathrm{Z}\mathrm{Z}+\mathrm {X}\rightarrow 2\mathrm{l} 2\nu +\mathrm {X}$$ and $$\mathrm {p}\mathrm {p}\rightarrow \mathrm {W}\mathrm{Z}+\mathrm {X}\rightarrow 3\mathrm{l} \nu +\mathrm {X}$$ processes are calculated using mcfm version 6.2 [3], and using PDFs from the Les Houches accord PDF (lhapdf) program, version 5.8.7 [30]. The PDF $$+$$ $$\alpha _\mathrm {S}$$ uncertainty in the $$\mathrm {W}$$$$\mathrm{Z}$$ cross section is evaluated as the maximum spread of the cross sections computed at $$\mu _{R} = \mu _{F} = m_{\mathbf {Z}}$$ with three PDF sets, including the corresponding uncertainties from one standard deviation variation of the PDF parameters and the $$\alpha _\mathrm {S}$$ value [31]. It is found to be 3.1 % (4.2 %) at 7 (8)$$~\mathrm{TeV}$$.

The uncertainty from the renormalization and factorization scales is evaluated as the maximum difference between the central value of the cross section at $$\mu _R = \mu _F = m_\mathrm{Z}$$ and the central values computed at $$\mu _R = \mu _F = m_\mathrm{Z}/2$$ and $$2\,m_\mathrm{Z}$$, using each of the three PDFs recommended in Ref. [31]. An uncertainty of 5.9 % (5.4 %) at 7 (8)$$~\mathrm{TeV}$$ is found for the $$\mathrm {W}$$$$\mathrm{Z}$$ background. For the $$\mathrm{Z}$$$$\mathrm{Z}$$ signal, we evaluate this theoretical uncertainty in the case of the exclusive production with 0 jets, to take into account the jet-veto applied in the signal selection, following the prescription described in Refs. [32, 33]. The exclusive cross section for $$\mathrm{Z}$$$$\mathrm{Z}$$ $$+$$ 0 jets is $$\sigma _{0j} = \sigma _{\ge 0j} - \sigma _{\ge 1j}$$, where $$\sigma _{\ge nj}$$ is the inclusive cross section of $$\mathrm{Z}$$$$\mathrm{Z}$$$$+$$ at least *n* jets, where $$n=0,1$$. According to Ref. [32], $$\sigma _{\ge 0j}$$ and $$\sigma _{\ge 1j}$$ are essentially uncorrelated, thus the uncertainty in $$\sigma _{0j}$$ can be computed as $$\epsilon _{0j} = \sqrt{{\epsilon _{\ge 0j}^2 + \epsilon _{\ge 1j}^2}}$$, where $$\epsilon _{\ge 0j}$$ and $$\epsilon _{\ge 1j}$$ are the uncertainties in $$\sigma _{\ge 0j}$$ and $$\sigma _{\ge 1j}$$, respectively. The cross sections are computed with mcfm, including the acceptance requirements on lepton $$p_{\mathrm {T}}$$ and $$\eta $$, charged dilepton mass, and $$E_{\mathrm{T}}^{\mathrm{miss}}$$, as well as the jet veto, when relevant. The cross section uncertainties are estimated by varying the renormalization and factorization scales, as explained above. Since the charged dilepton $$p_{\mathrm {T}}$$ spectrum is the observable from which limits on ATGCs are derived, the uncertainty in $$\sigma _{0j}$$ is computed in different intervals of charged dilepton $$p_{\mathrm {T}}$$.

The uncertainty in the NLO EW correction to $$\mathrm{Z}$$$$\mathrm{Z}$$ production, corresponding to missing higher-order terms in the computation, is estimated as the product of the NLO QCD and EW corrections [7]. The uncertainty in the EW correction to $$\mathrm {W}$$$$\mathrm{Z}$$ production is estimated as 100 % of the correction, to account for the poorly known fraction of photon $$+$$ quark-induced events [8] passing the jet veto.

### Acceptance

The kinematic acceptance for the signal is computed using mcfm. Kinematic requirements, based on those used in the signal selection, are applied to the charged leptons and neutrinos at the generator level. The acceptance is determined by comparing the cross sections with and without the kinematic requirements. The systematic uncertainty is evaluated as the variation in the acceptance resulting from varying the renormalization and factorization scales from $m_\mathrm{Z}$ to $m_\mathrm{Z}/2$ and $2\,m_\mathrm{Z}$, summed in quadrature with the variation obtained from using different PDF sets and from varying the PDF parameters and the $\alpha _\mathrm {S}$ value by one standard deviation. The result is 2.8 % at both 7 and 8$~\mathrm{TeV}$.

### Luminosity

The uncertainty in the luminosity measurement is 2.2 % in 2011, and 2.6 % in 2012 [34].

### Lepton trigger and identification efficiency

Lepton trigger and identification efficiencies are determined from data, using the tag-and-probe technique with $$\mathrm{Z}\ \rightarrow \mathrm{l} \mathrm{l} $$ events [35], and used to correct the simulated samples. The total uncertainty in the lepton efficiency amounts to about 3 % for $$\mathrm {e}$$$$\mathrm {e}$$ events, and 4 % for $$\mathrm {\mu }$$$$\mathrm {\mu }$$ events.

### Lepton momentum scale

The systematic uncertainty in the lepton momentum scale is computed by shifting the nominal momenta by $$\pm 1\sigma $$ and propagating the variations to the reduced $$E_{\mathrm{T}}^{\mathrm{miss}}$$. We assume an uncertainty of 2 % (3.5 %) in the energy of electrons reconstructed in the ECAL barrel (endcap), and 1 % in the muon momentum. The resulting variations of the final yields are 2.5 % for the $$\mathrm {e}$$$$\mathrm {e}$$ channel, and 1.0 % for the $$\mathrm {\mu }$$$$\mathrm {\mu }$$ channel and they are treated as a shape uncertainty.

### Jet energy scale and resolution

The uncertainty in the calibration of the jet energy scale directly affects the jet veto, the calculation of reduced $$E_{\mathrm{T}}^{\mathrm{miss}}$$, and the selection of the balance variable. The JES uncertainty is estimated by shifting the jet energies by $$\pm 1\sigma $$ and propagating the variations to the reduced $$E_{\mathrm{T}}^{\mathrm{miss}}$$ and all the other relevant observables. Uncertainties in the final yields of 3–4 (7–8) % are found for both the $$\mathrm {e}$$$$\mathrm {e}$$ and $$\mathrm {\mu }$$$$\mathrm {\mu }$$ final states at 7 (8)$$~\mathrm{TeV}$$.

Similarly, a systematic uncertainty in jet energy resolution (JER) is computed. As explained above, the energy of jets in simulation is corrected to reproduce the resolution observed in data. Such corrections are varied according to their uncertainties and these variations are propagated to all the observables and selections dependent on jet energy. An uncertainty in the final yields of less than 1 % is found in both $$\mathrm {e}$$$$\mathrm {e}$$ and $$\mathrm {\mu }$$$$\mathrm {\mu }$$ final states: 0.4 % (0.8 %) at 7 (8)$$~\mathrm{TeV}$$.

**Fig. 2:**
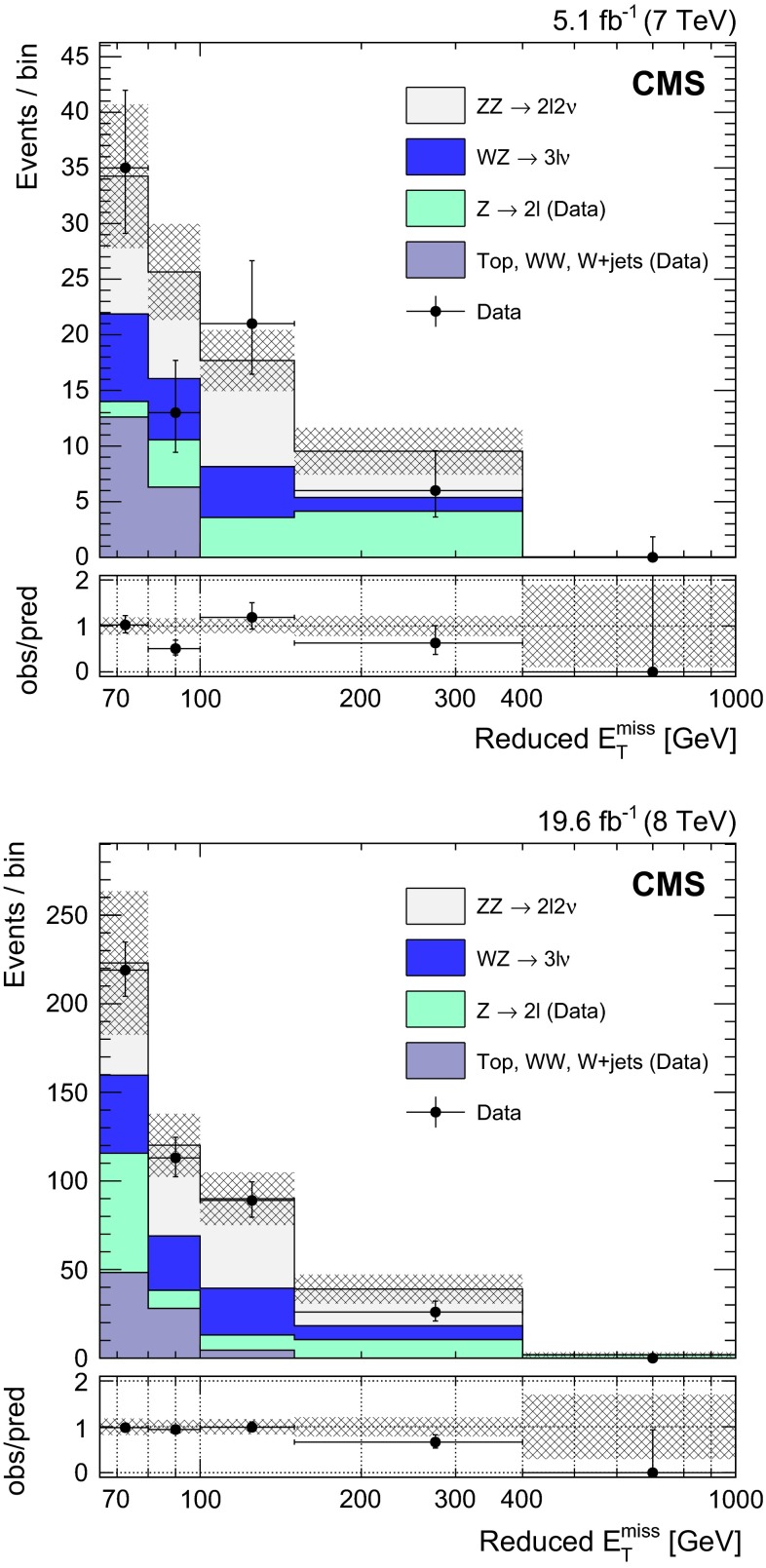
Reduced $E_{\mathrm{T}}^{\mathrm{miss}}$ distribution in $\mathrm{l} \mathrm{l}$ ($\mathrm{l} = \mathrm {e},\mathrm {\mu }$) channels, after the full selection, at 7$~\mathrm{TeV}$ (*top*) and 8$~\mathrm{TeV}$ (*bottom*). The DY and $\mathrm {W}$ $\mathrm {W}$, $\mathrm {W}$ $+$ jets, and top backgrounds are estimated with data-driven methods. The *gray error band* includes statistical and systematic uncertainties in the predicted yields. In the *bottom plots*, *vertical error bars* and *bands* are relative to the total predicted yields. In *all* plots, *horizontal error bars* indicate the bin width

**Table 2:** Predicted signal and background yields at 7 and 8$~\mathrm{TeV}$, and corresponding values obtained from the combined maximum likelihood fit to the $\mathrm {e}$ $\mathrm {e}$ and $\mathrm {\mu }$ $\mathrm {\mu }$ channels. The uncertainties include both the statistical and systematic components

Dataset	Process	Channel	Predicted yield	Fitted yield	Observed
7$~\mathrm{TeV}$	$\mathrm{Z}\mathrm{Z}\rightarrow 2\mathrm{l} 2\nu$	$\mathrm {e}$ $\mathrm {e}$	$14.0 \pm 1.9$	$12.0 \pm 4.4$	$-$
		$\mathrm {\mu }$ $\mathrm {\mu }$	$21.7 \pm 3.2$	$18.4 \pm 6.8$	$-$
	$\mathrm {W}\mathrm{Z}\rightarrow 3\mathrm{l} \nu$	$\mathrm {e}$ $\mathrm {e}$	$7.7 \pm 0.9$	$7.9 \pm 1.0$	$-$
		$\mathrm {\mu }$ $\mathrm {\mu }$	$11.5 \pm 1.6$	$11.6 \pm 1.2$	$-$
	$\mathrm{Z}$ + jets	$\mathrm {e}$ $\mathrm {e}$	$5.0 \pm 2.7$	$4.8 \pm 2.3$	$-$
		$\mathrm {\mu }$ $\mathrm {\mu }$	$8.3 \pm 4.8$	$4.8 \pm 3.0$	$-$
	Nonresonant	$\mathrm {e}$ $\mathrm {e}$	$7.7 \pm 3.1$	$7.4 \pm 2.3$	$-$
		$\mathrm {\mu }$ $\mathrm {\mu }$	$11.2 \pm 4.8$	$9.2 \pm 3.1$	$-$
	Total	$\mathrm {e}$ $\mathrm {e}$	$34.4 \pm 6.2$	$32.1 \pm 3.9$	35
		$\mathrm {\mu }$ $\mathrm {\mu }$	$52.7 \pm 9.7$	$44.0 \pm 5.3$	40
8$~\mathrm{TeV}$	$\mathrm{Z}\mathrm{Z}\rightarrow 2\mathrm{l} 2\nu$	$\mathrm {e}$ $\mathrm {e}$	$77 \pm 16$	$69 \pm 13$	$-$
		$\mathrm {\mu }$ $\mathrm {\mu }$	$109 \pm 23$	$100 \pm 19$	$-$
	$\mathrm {W}\mathrm{Z}\rightarrow 3\mathrm{l} \nu$	$\mathrm {e}$ $\mathrm {e}$	$45 \pm 6$	$43.9 \pm 5.6$	$-$
		$\mathrm {\mu }$ $\mathrm {\mu }$	$64 \pm 8$	$63.8 \pm 7.3$	$-$
	$\mathrm{Z}$ + jets	$\mathrm {e}$ $\mathrm {e}$	$36 \pm 12$	$27.7 \pm 7.9$	$-$
		$\mathrm {\mu }$ $\mathrm {\mu }$	$63 \pm 21$	$52 \pm 14$	$-$
	Nonresonant	$\mathrm {e}$ $\mathrm {e}$	$31 \pm 9$	$34.1 \pm 7.2$	$-$
		$\mathrm {\mu }$ $\mathrm {\mu }$	$50 \pm 14$	$54 \pm 12$	$-$
	Total	$\mathrm {e}$ $\mathrm {e}$	$189 \pm 31$	$174.7 \pm 10$	176
		$\mathrm {\mu }$ $\mathrm {\mu }$	$286 \pm 49$	$269.8 \pm 15$	271

Since the shapes of the distributions are expected to be affected by variations in the JES and the JER, these sources are treated as shape uncertainties in the extraction of the cross section.

### $\mathrm{b}$-jet veto

The $\mathrm{b}$-tagging efficiency is taken from Ref. [36]. In simulation, the nominal working point for this $\mathrm{b}$-tagger is shifted to reproduce the efficiency observed in data. The uncertainty in the measured efficiency is propagated to the event yields of the processes estimated from simulation by applying further shifts to the discriminator threshold. A very small uncertainty in the final yields of the MC samples is found: 0.1–0.15 % at both 7 and 8$~\mathrm{TeV}$.

### Pileup

Simulated samples are reweighted to reproduce the pileup conditions observed in data. To compute the uncertainty related to this procedure, we shift the number of interactions by 8 % when reweighting the simulated samples. The variation of the final yields induced by this procedure is less than 1 % in $$\mathrm{Z}$$$$\mathrm{Z}$$ and $$\mathrm {W}$$$$\mathrm{Z}$$ processes. However, the shapes of the kinematic distributions can vary in this procedure, so the varied distributions are used as shape uncertainties in the cross section fit.

**Table 3:** Cross sections (fb) for process $\mathrm {p}\mathrm {p}\rightarrow \mathrm{Z}\mathrm{Z}\rightarrow 2\mathrm{l} 2\nu$ (where $\mathrm{l}$ denotes a charged lepton of a given flavor, $\nu$ a neutrino of any flavor) at 7 and 8$~\mathrm{TeV}$, with both $\mathrm{Z}$ boson masses in the range 60–120$\,\text {GeV}$, measured in the $\mathrm {e}$ $\mathrm {e}$ and $\mathrm {\mu }$ $\mathrm {\mu }$ channels and the two channels combined

Channel	$\sqrt{s} = 7~\mathrm{TeV}$	$\sqrt{s} = 8~\mathrm{TeV}$
$\mathrm {e}$ $\mathrm {e}$	$98_{-31}^{+35}\,\text {(stat)} \,_{-22}^{+27}\,\text {(syst)} \pm 2\,\text {(lumi)}$	$83_{-16}^{+17}\,\text {(stat)} \,_{-19}^{+26}\,\text {(syst)} \pm 2\,\text {(lumi)}$
$\mathrm {\mu }$ $\mathrm {\mu }$	$47_{-21}^{+24}\,\text {(stat)} \,_{-19}^{+20}\,\text {(syst)} \pm 1\,\text {(lumi)}$	$98_{-14}^{+14}\,\text {(stat)} \,_{-22}^{+29}\,\text {(syst)} \pm 3\,\text {(lumi)}$
Combined	$66_{-18}^{+20}\,\text {(stat)} \,_{-14}^{+18}\,\text {(syst)} \pm 1\,\text {(lumi)}$	$92_{-10}^{+11}\,\text {(stat)} \,_{-19}^{+25}\,\text {(syst)} \pm 2\,\text {(lumi)}$
Theory	$79^{+4}_{-3}\,\text {(theo)}$	$97^{+4}_{-3}$ $\,\text {(theo)}$

**Table 4:** Systematic uncertainties in the cross sections due to each source separately, after the maximum likelihood fit to extract the $\mathrm{Z}$ $\mathrm{Z}$ cross section. The uncertainties marked with an asterisk ($*$) are used as shape uncertainties in the fit

Source of uncertainty	Uncertainty (%)	
	7$~\mathrm{TeV}$	8$~\mathrm{TeV}$
($*$) MC statistics: $\mathrm{Z}$ $\mathrm{Z}$ ($\mathrm {e}$ $\mathrm {e}$ channel)	0.8	1.0
($*$) MC statistics: $\mathrm{Z}$ $\mathrm{Z}$ ($\mathrm {\mu }$ $\mathrm {\mu }$ channel)	1.3	1.1
($*$) MC statistics: $\mathrm {W}$ $\mathrm{Z}$ ($\mathrm {e}$ $\mathrm {e}$ channel)	1.7	0.9
($*$) MC statistics: $\mathrm {W}$ $\mathrm{Z}$ ($\mathrm {\mu }$ $\mathrm {\mu }$ channel)	1.7	1.0
($*$) Control sample statistics: DY ($\mathrm {e}$ $\mathrm {e}$ channel)	6.9	2.3
($*$) Control sample statistics: DY ($\mathrm {\mu }$ $\mathrm {\mu }$ channel)	5.8	4.9
($*$) Control sample statistics: NRB ($\mathrm {e}$ $\mathrm {e}$ channel)	6.3	3.0
($*$) Control sample statistics: NRB ($\mathrm {\mu }$ $\mathrm {\mu }$ channel)	8.1	4.4
$\mathrm {W}$ $\mathrm{Z}$ cross section: PDF + $\alpha _{\mathrm S}$	1.9	2.6
($*$) $\mathrm{Z}$ $\mathrm{Z}$ + $\mathrm {W}$ $\mathrm{Z}$ cross section: scales	17	16
($*$) $\mathrm{Z}$ $\mathrm{Z}$ + $\mathrm {W}$ $\mathrm{Z}$ cross section: NLO EW corr.	2.4	2.3
Signal acceptance	2.8	2.8
($*$) Pileup	0.5	1.1
Muon trigger, ID, isolation	4.1	3.6
Electron trigger, ID, isolation	1.7	2.0
($*$) Lepton momentum scale	2.6	3.7
($*$) JES	6.0	12
($*$) JER	0.8	1.4
($*$) Unclustered $E_{\mathrm{T}}^{\mathrm{miss}}$	2.1	3.2
($*$) $\mathrm{b}$-jet veto	0.3	0.5
Drell–Yan bkg. normalization	6.6	8.4
Top-quark and $\mathrm {W}$ $\mathrm {W}$ bkg. normalization	7.7	7.1
Total systematic uncertainty	24.6	23.5
Statistical uncertainty	28.0	11.9

### Drell–Yan

The uncertainty in the DY contribution is propagated from the uncertainty in the reweighted photon spectrum that is used in the estimate of DY background from data, and is dominated by the subtraction of backgrounds due to EW processes. As explained in Sect. 5, the DY background estimate is assigned an uncertainty of 25 % (40 %) at 7 (8)$~\mathrm{TeV}$, evaluated from the relative difference between dilepton yields in data and simulation in a control region.

### Top-quark and $$\mathrm {W}$$$$\mathrm {W}$$ backgrounds

The uncertainty in the estimate of the NRB is derived from the statistical uncertainties in the scale factors in Eq. (1), and from a closure test of the data-driven method for the measurement of this background performed on simulated data. It is found to be about 20 % at both 7 and 8$~\mathrm{TeV}$.

## Measurement of the $$\mathrm{Z}$$$$\mathrm{Z}$$ production cross section

We extract the $$\mathrm{Z}$$$$\mathrm{Z}$$ production cross section using a profile likelihood fit [37] to the reduced-$$E_{\mathrm{T}}^{\mathrm{miss}}$$ distribution, shown in Fig. 2. The fit takes into account the expectations for the different background processes and the $$\mathrm{Z}$$$$\mathrm{Z}$$ signal. Each systematic uncertainty is introduced in the fit as a nuisance parameter with a log-normal prior. For the signal we consider a further multiplicative factor, which is the ratio of the cross section measured in data to the expected theoretical value, i.e. the signal strength $$\mu =\sigma /\sigma _\text {th}$$. Maximizing the profile likelihood, we obtain the $$\mathrm{Z}$$$$\mathrm{Z}$$ production cross section from the signal strength parameter, as well as optimal fits of the background yields by varying nuisance parameters within their constraints. Table 2 shows the expected signal and background yields, and the corresponding values after the combined fit to the $$\mathrm {e}$$$$\mathrm {e}$$ and $$\mathrm {\mu }$$$$\mathrm {\mu }$$ channels. The uncertainties include both the statistical and systematic components.

The cross sections are extracted from individual fits to the $$\mathrm {e}$$$$\mathrm {e}$$ and $$\mathrm {\mu }$$$$\mathrm {\mu }$$ channels and from a simultaneous fit to both channels. Table 3 reports the measured $$\mathrm {p}\mathrm {p}\rightarrow \mathrm{Z}\mathrm{Z}\rightarrow 2\mathrm{l} 2\nu $$ exclusive cross section, i.e. the production cross section of $$\mathrm{Z}$$$$\mathrm{Z}$$ pairs with mass $$60<M_\mathrm{Z}< 120\,\text {GeV} $$, with no restrictions on lepton acceptance nor jet number, times the branching fraction to final states with two charged leptons of a given flavor and two neutrinos of any flavor. This is obtained by rescaling the theoretical prediction for the exclusive cross section in the same kinematic range by the fitted signal strength. These theoretical predictions are computed at NLO in QCD with mcfm and corrected for NLO EW effects: $$79^{+4}_{-3}$$ ($$97^{+4}_{-3}$$)$$\text {~fb}$$ at 7 (8)$$~\mathrm{TeV}$$.

The measured inclusive $$\mathrm{Z}$$$$\mathrm{Z}$$ cross section is obtained by rescaling the theoretical inclusive cross section computed in the zero-width approximation [3] and corrected for NLO EW effects [7] (see Sect. 1), by the same fitted signal strength. This procedure properly accounts for the contribution of virtual photon decays to the charged-lepton pair production, and yields a measured cross section that can be compared directly with theoretical calculations of inclusive pure $$\mathrm{Z}$$$$\mathrm{Z}$$ production in the zero-width approximation. The results are:

$$\begin{aligned}&7~\mathrm{TeV} :\\&\quad \sigma (\mathrm {p}\mathrm {p}\rightarrow \mathrm{Z}\mathrm{Z}) = 5.1_{-1.4}^{+1.5}\,\text {(stat)} \,_{-1.1}^{+1.4}\,\text {(syst)} \pm 0.1\,\text {(lumi)} \text {~pb}, \end{aligned}$$

$$\begin{aligned}&8~\mathrm{TeV} :\\&\quad \sigma (\mathrm {p}\mathrm {p}\rightarrow \mathrm{Z}\mathrm{Z}) = 7.2_{-0.8}^{+0.8}\,\text {(stat)} \,_{-1.5}^{+1.9}\,\text {(syst)} \pm 0.2\,\text {(lumi)} \text {~pb}. \end{aligned}$$

This is the first cross section measurement in the $2\mathrm{l} 2\nu$ channel at 8 TeV. The measurements are less than one standard deviation from the SM predictions at both 7 and 8$~\mathrm{TeV}$. The uncertainties are approximately twice as large as those from the CMS measurement in the $4\ell$ channel [10, 11], and the channels agree within uncertainties.

The *p*-values of the simultaneous fit to the $$\mathrm {e}$$$$\mathrm {e}$$ and $$\mathrm {\mu }$$$$\mathrm {\mu }$$ channels are 0.335 (0.569) at 7 (8)$$~\mathrm{TeV}$$. The data are also consistent with the reduced $$E_{\mathrm{T}}^{\mathrm{miss}}$$ spectra uncorrected for NLO EW effects, but with slightly smaller *p*-values of 0.322 (0.477) at 7 (8)$$~\mathrm{TeV}$$. The application of EW corrections thus improves the modeling of the diboson processes and leads to a better agreement between the simulated and observed spectra.

Table 4 shows a summary of the sources of systematic uncertainty described in Sect. 6, with the corresponding contributions to the total systematic uncertainty in the cross sections.

**Fig. 3:**
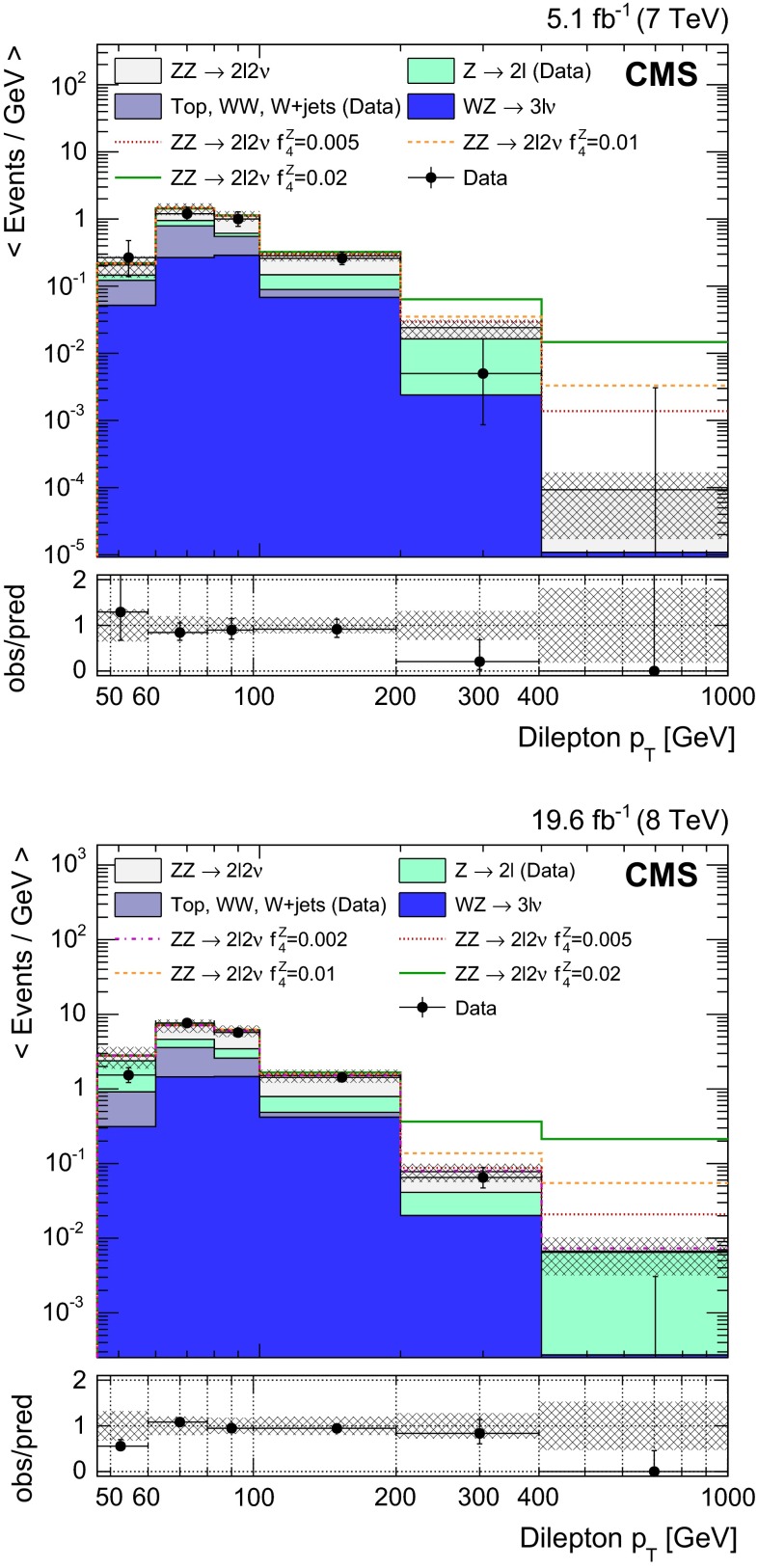
Dilepton ($\mathrm{l} = \mathrm {e}, \mathrm {\mu }$) transverse momentum distributions at 7$~\mathrm{TeV}$ (*top*) and 8$~\mathrm{TeV}$ (*bottom*). The DY and $\mathrm {W}$ $\mathrm {W}$, $\mathrm {W}$ $+$ jets, and top backgrounds are estimated from control samples in data. The *gray error band* includes statistical and systematic uncertainties in the predicted yields. In the *bottom plots*, *vertical error bars* and *bands* are relative to the total predicted yields. In *all* plots, *horizontal error bars* indicate the bin width

## Anomalous couplings

The existence of neutral trilinear gauge couplings is forbidden at the tree level, but allowed in some extensions of the SM [38]. The $$\mathrm{Z}$$$$\mathrm{Z}$$ production process provides a way to probe the existence of such anomalous couplings at the $$\mathrm{Z}$$$$\mathrm{Z}$$$$\mathrm{Z}$$ and $$\gamma $$$$\mathrm{Z}$$$$\mathrm{Z}$$ vertices.

Neutral couplings $\mathrm {V}^{(*)}\mathrm{Z}\mathrm{Z}$ ($\mathrm {V} = \mathrm{Z}, \gamma$) can be described using the following effective Lagrangian [39]:

3 $$\begin{aligned} \mathcal {L}_{\mathrm {V}\mathrm{Z}\mathrm{Z}}= & {} -\frac{e}{M_{\mathbf {Z}}^2} \Big \{ [f_4^\gamma (\partial _\mu F^{\mu \alpha }) +f_4^\mathrm{Z}(\partial _\mu Z^{\mu \alpha })] Z_\beta (\partial ^\beta Z_\alpha )\nonumber \\&-[f_5^\gamma (\partial ^\mu F_{\mu \alpha }) +f_5^\mathrm{Z}(\partial ^\mu Z_{\mu \alpha })] \tilde{Z}^{\alpha \beta }Z_\beta \Big \}, \end{aligned}$$

where *Z* represents the $\mathrm{Z}$ boson and $F_{\mu \alpha }$ represents the electromagnetic field tensor. The coefficients $f_i^\gamma$ and $f_i^\mathrm{Z}$ correspond to couplings $\gamma ^{(*)}\mathrm{Z}\mathrm{Z}$ and $\mathrm{Z}^{(*)}\mathrm{Z}\mathrm{Z}$, respectively. All the operators in Eq. (3) are Lorentz-invariant and $\mathrm {U}(1)_{\mathrm {EM}}$ gauge-invariant, but not invariant under $\mathrm {SU}(2)_{\mathrm {L}}\times \mathrm {U}(1)_{\mathrm {Y}}$ gauge symmetry. The terms corresponding to $f_4^\mathrm {V}$ parameters violate the CP symmetry, while the terms corresponding to $f_5^\mathrm {V}$ parameters conserve CP.

**Table 5:** Summary of 95 % CL intervals for the neutral ATGC coefficients, set by the $2\mathrm{l} 2\nu$ final states using the 7 and 8$~\mathrm{TeV}$ CMS datasets. The expected 95 % CL intervals obtained using the 7 and 8$~\mathrm{TeV}$ simulated samples are also shown. No form factor is used

Dataset	$f_4^\mathrm{Z}$	$f_4^{\gamma }$	$f_5^\mathrm{Z}$	$f_5^{\gamma }$
7$~\mathrm{TeV}$	[$-$0.010; 0.011]	[$-$0.012; 0.013]	[$-$0.010; 0.010]	[$-$0.013; 0.013]
8$~\mathrm{TeV}$	[$-$0.0033; 0.0037]	[$-$0.0044; 0.0038]	[$-$0.0033; 0.0035]	[$-$0.0039; 0.0043]
Combined	[$-$0.0028; 0.0032]	[$-$0.0037; 0.0033]	[$-$0.0029; 0.0031]	[$-$0.0033; 0.0037]
Expected (7 and 8$~\mathrm{TeV}$)	[$-$0.0048; 0.0051]	[$-$0.0060; 0.0053]	[$-$0.0048; 0.0050]	[$-$0.0057; 0.0062]

**Table 6:** Summary of 95 % CL intervals for the neutral ATGC coefficients, set by the combined analysis of $4\mathrm{l}$ and $2\mathrm{l} 2\nu$ final states. The intervals obtained separately by the two analyses using the 7 and 8$~\mathrm{TeV}$ CMS data sets are shown, as well as their combination. The expected 95 % CL intervals obtained using the 7 and 8$~\mathrm{TeV}$ simulated samples of both analyses are also shown. No form factor is used

Dataset	$f_4^\mathrm{Z}$	$f_4^\gamma$	$f_5^\mathrm{Z}$	$f_5^\gamma$
7$~\mathrm{TeV}$, $4\mathrm{l}$	[$-$0.010; 0.011]	[$-$0.012; 0.013]	[$-$0.011; 0.011]	[$-$0.013; 0.013]
7$~\mathrm{TeV}$, $2\mathrm{l} 2\nu$	[$-$0.010; 0.011]	[$-$0.012; 0.013]	[$-$0.010; 0.010]	[$-$0.013; 0.013]
8$~\mathrm{TeV}$, $4\mathrm{l}$	[$-$0.0041; 0.0044]	[$-$0.0052; 0.0048]	[$-$0.0041; 0.0040]	[$-$0.0048; 0.0045]
8$~\mathrm{TeV}$, $2\mathrm{l} 2\nu$	[$-$0.0033; 0.0037]	[$-$0.0044; 0.0038]	[$-$0.0033; 0.0035]	[$-$0.0039; 0.0043]
Combined	[$-$0.0022; 0.0026]	[$-$0.0029; 0.0026]	[$-$0.0023; 0.0023]	[$-$0.0026; 0.0027]
Expected	[$-$0.0036; 0.0039]	[$-$0.0046; 0.0041]	[$-$0.0036; 0.0037]	[$-$0.0043; 0.0043]
($4\mathrm{l}$ and $2\mathrm{l} 2\nu$, 7 and 8$~\mathrm{TeV}$)				

To avoid unitarity violation at energies above the scale ($\Lambda$) of new physics, the Lagrangian of Eq. (3) can be modified with form factors of the type $1{/\!}\left( 1+\hat{s}/\Lambda \right) ^n$, where $\sqrt{\hat{s}}$ is the effective center-of-mass energy of the collision. No form-factor scaling is used in this analysis. This allows to provide results without any bias that can arise due to a particular choice of the form-factor energy dependence.

Previous studies of neutral anomalous triple gauge couplings were performed at LEP2 [40], Tevatron [41], and LHC [9–11]. No deviation from the SM expectation has been observed so far, and the best limits were set by the LHC measurements based on integrated luminosities of about 5 (19.6)$\,\text {fb}^\text {-1}$ at 7 (8)$~\mathrm{TeV}$.

### Limits from the $\mathrm{Z}\mathrm{Z}\rightarrow 2\mathrm{l} 2\nu$ channel

In the following, we extract limits on the neutral triple gauge couplings $$\mathrm {V}^{(*)}\mathrm{Z}\mathrm{Z}$$ with the same datasets at 7 and 8$$~\mathrm{TeV}$$ as used for the $$\mathrm{Z}$$$$\mathrm{Z}$$ cross section measurement described in the previous section. Limits on the four $$f_i^\mathrm {V}$$ parameters are set by comparing the data with theoretical predictions.

Figure 3 shows the charged dilepton $$p_{\mathrm {T}}$$ distribution after the full selection described in Table 1, in data and simulation, including sherpa samples with different values of the $$f_4^\mathrm{Z}$$ parameter. The contribution from the anomalous couplings enhances the high-$$p_{\mathrm {T}}$$ region of the distribution. The charged dilepton $$p_{\mathrm {T}}$$ is thus a good observable to probe for the presence of ATGCs. The DY and nonresonant backgrounds are estimated from data as described above. The SM $$\mathrm{Z}$$$$\mathrm{Z}$$ process is simulated here using the MadGraph sample described in Sect. 2, with NLO QCD corrections computed with mcfm and NLO EW corrections from Ref. [7]. The contribution of the ATGCs is obtained from the sherpa samples mentioned above, by subtracting the SM sherpa contribution to the charged dilepton $$p_{\mathrm {T}}$$, and is summed to the MadGraph$$\mathrm{Z}$$$$\mathrm{Z}$$ distribution. The interference of the ATGC signal and the SM $$\mathrm{Z}$$$$\mathrm{Z}$$ production is included, except for $$p_{\mathrm {T}} (\mathrm{Z})<200\,\text {GeV} $$, which has a negligible impact on the limits. The expected signal yields in each $$p_{\mathrm {T}}$$ bin are interpolated between different values of the ATGC coupling parameters using a second-degree polynomial, since the signal cross section depends quadratically on such parameters.

The limits are calculated with a profile likelihood method. We set one-dimensional limits on the four parameters, i.e. varying independently a single parameter at a time, while fixing the other three to zero. The 95 % CL one-dimensional limits on the four parameters are reported in Table 5 for 7$~\mathrm{TeV}$, 8$~\mathrm{TeV}$, and combined datasets. The observed exclusion limits are about one standard deviation tighter than the expected ones, which is attributed primarily to the observed deficit of events in the highest bin of dilepton $p_{\mathrm {T}}$. The limits set are of comparable sensitivity to those previously obtained by CMS in the $4\mathrm{l}$ channel [10, 11].

### Combined limits from the $\mathrm{Z}\mathrm{Z}\rightarrow 4\mathrm{l}$ and $\rightarrow 2\mathrm{l} 2\nu$ channels

We proceed with the combination of the results of the previously published $\mathrm{Z}\mathrm{Z}\rightarrow 4\mathrm{l}$ analyses [10, 11] with the present results. In doing this, the published analysis of the $4\mathrm{l}$ ($\mathrm{l} =\mathrm {e},\mathrm {\mu }$) channel is unchanged, except that NLO EW corrections to the SM $\mathrm{Z}\mathrm{Z}\rightarrow 4\mathrm{l}$ background are included in the same way as in the present analysis. We use a profile likelihood method to calculate the 95 % CL one-dimensional intervals for the four parameters, combining the data in the $4\mathrm{l}$ and $2\mathrm{l} 2\nu$ channels, at 7 and 8$~\mathrm{TeV}$. The systematic uncertainties in the signal and diboson background cross sections, in the integrated luminosity, and in the lepton efficiencies are treated as fully correlated between the two channels. Table 6 shows the intervals obtained by combining the four separate data sets. The combined analysis improves the sensitivity of the two separate channels, and the limits are more stringent than all the results published to date.

## Summary

We have measured the $$\mathrm{Z}$$$$\mathrm{Z}$$ production cross section in the $$2\mathrm{l} 2\nu $$ channel in proton–proton collisions at center-of-mass energies of 7 and 8$$~\mathrm{TeV}$$. The data samples selected for the study correspond to an integrated luminosity of 5.1 (19.6)$$\,\text {fb}^\text {-1}$$ at 7 (8)$$~\mathrm{TeV}$$. We have measured

$$\begin{aligned} \sigma (\mathrm {p}\mathrm {p}\rightarrow \mathrm{Z}\mathrm{Z}) = 5.1_{-1.4}^{+1.5}\,\text {(stat)} \,_{-1.1}^{+1.4}\,\text {(syst)} \pm 0.1\,\text {(lumi)} \text {~pb} \end{aligned}$$

at 7$$~\mathrm{TeV}$$, and

$$\begin{aligned} \sigma (\mathrm {p}\mathrm {p}\rightarrow \mathrm{Z}\mathrm{Z}) = 7.2_{-0.8}^{+0.8}\,\text {(stat)} \,_{-1.5}^{+1.9}\,\text {(syst)} \pm 0.2\,\text {(lumi)} \text {~pb} \end{aligned}$$

at 8$$~\mathrm{TeV}$$, in agreement with theory calculations, $$6.2^{+0.3}_{-0.2}$$$$\text {~pb}$$ ($$7.6^{+0.4}_{-0.3}$$$$\text {~pb}$$) at 7 (8)$$~\mathrm{TeV}$$, which include NLO QCD corrections [3] and NLO EW corrections [7, 8]. The selected data have also been analyzed to search for ATGCs involving the $$\mathrm{Z}$$$$\mathrm{Z}$$ final state. In the absence of any observation of new physics, we have set the most stringent limits to date on the relevant ATGC parameters. In addition, by combining the selected data with the CMS data for the four-charged-lepton final state we have set even tighter constraints.
